# A hybrid TIM complex mediates protein import into hydrogenosomes of *Trichomonas vaginalis*

**DOI:** 10.1186/s12915-024-01928-8

**Published:** 2024-06-03

**Authors:** Abhijith Makki, Sami Kereïche, Tien Le, Jitka Kučerová, Petr Rada, Vojtěch Žárský, Ivan Hrdý, Jan Tachezy

**Affiliations:** 1 Department of Parasitology, Faculty of Science, Charles University, BIOCEV, Průmyslová 595, 25250 Vestec, Czech Republic; 2 Institute of Biology and Medical Genetics, First Faculty of Medicine, Charles University, Albertov 4, 12800 Prague 2, Czech Republic; 3 Department of Biology and Ecology, Faculty of Science, University of Ostrava, Ostrava, Czech Republic; 4 Present address: Department of Cellular Biochemistry, University Medical Center Göttingen, D-37073 Göttingen, Germany

**Keywords:** Hydrogenosomes, Mitochondria, Parasite, Presequence translocase-associated motor, Protein import machinery, TIM22 complex, TIM23 complex, *Trichomonas vaginalis*

## Abstract

**Background:**

Hydrogenosomes are a specific type of mitochondria that have adapted for life under anaerobiosis. Limited availability of oxygen has resulted in the loss of the membrane-associated respiratory chain, and consequently in the generation of minimal inner membrane potential (Δψ), and inefficient ATP synthesis via substrate-level phosphorylation. The changes in energy metabolism are directly linked with the organelle biogenesis. In mitochondria, proteins are imported across the outer membrane via the Translocase of the Outer Membrane (TOM complex), while two Translocases of the Inner Membrane, TIM22, and TIM23, facilitate import to the inner membrane and matrix. TIM23-mediated steps are entirely dependent on Δψ and ATP hydrolysis, while TIM22 requires only Δψ. The character of the hydrogenosomal inner membrane translocase and the mechanism of translocation is currently unknown.

**Results:**

We report unprecedented modification of TIM in hydrogenosomes of the human parasite *Trichomonas vaginalis* (TvTIM). We show that the import of the presequence-containing protein into the hydrogenosomal matrix is mediated by the hybrid TIM22-TIM23 complex that includes three highly divergent core components, TvTim22, TvTim23, and TvTim17-like proteins. The hybrid character of the TvTIM is underlined by the presence of both TvTim22 and TvTim17/23, association with small Tim chaperones (Tim9-10), which in mitochondria are known to facilitate the transfer of substrates to the TIM22 complex, and the coupling with TIM23-specific ATP-dependent presequence translocase-associated motor (PAM). Interactome reconstruction based on co-immunoprecipitation (coIP) and mass spectrometry revealed that hybrid TvTIM is formed with the compositional variations of paralogs. Single-particle electron microscopy for the 132-kDa purified TvTIM revealed the presence of a single ring of small Tims complex, while mitochondrial TIM22 complex bears twin small Tims hexamer. TvTIM is currently the only TIM visualized outside of Opisthokonta, which raised the question of which form is prevailing across eukaryotes. The tight association of the hybrid TvTIM with ADP/ATP carriers (AAC) suggests that AAC may directly supply ATP for the protein import since ATP synthesis is limited in hydrogenosomes.

**Conclusions:**

The hybrid TvTIM in hydrogenosomes represents an original structural solution that evolved for protein import when Δψ is negligible and remarkable example of evolutionary adaptation to an anaerobic lifestyle.

**Supplementary Information:**

The online version contains supplementary material available at 10.1186/s12915-024-01928-8.

## Background

Mitochondrial biogenesis and functions depend on the import of proteins that are synthesized by the cytosolic ribosomes. Proteins in the inner mitochondrial membrane and matrix, constituting around 75% of the mitochondrial proteome, are initially imported via the Translocase of the Outer Membrane (TOM complex) [1, 2]. Then, proteins with a cleavable N-terminal sequence (NTS, syn. presequence) are transferred to the Translocase of the Inner Membrane 23 (TIM23 complex), while multispanning proteins such as metabolite carrier proteins (MCPs) are directed to the TIM22 complex [3, 4]. The TIM23 complex couples to an ATP-powered presequence translocase-associated motor (PAM) to import proteins into the mitochondrial matrix [4, 5]. In yeast and humans, the TIM23 complex consists of membrane-embedded protein-conducting subunits Tim17 and Tim23, receptor Tim50, Mgr2/ROMO1, and Tim21, and PAM consists of Tim44, ATP-hydrolyzing Hsp70, Pam18, Pam16, and Mge1 [4–10]. In yeast, the TIM22 complex consists of four membrane-integrated subunits, core Tim22, receptor Tim54, Tim18, and Sdh3 associated with the hexameric Tim9-10-12 chaperone complex in the IMS [11]. In the human TIM22 complex, Tim18 and Sdh3 are absent, but there is a specific subunit Tim29 [12, 13]. During the transfer of substrates from the TOM complex to the TIM22 complex, small Tim chaperones—essential Tim9 and Tim10 complex, or non-essential Tim8-Tim13 complex bind to the hydrophobic MCPs, thereby preventing their aggregation in the intermembrane space (IMS) [11, 14, 15]. Tim17, Tim22, and Tim23 belong to the Tim17 family of proteins, derived from a single ancestral protein and were likely present in the last eukaryotic ancestor (LECA) [10]. However, in several unicellular eukaryotes, the spectrum of Tim17 family proteins was secondarily reduced to a single type [16]. For example, *Trypanosoma brucei* contains a single aerobic mitochondrion, which houses a Tim22-based divergent TIM complex that can both import matrix proteins and insert proteins into the inner membrane [17].

With over 150 million cases every year globally, trichomoniasis is the most common non-viral sexually transmitted infection, caused by a parasite protist of the Parabasalia group, *Trichomonas vaginalis* [18]. *T. vaginalis* has an anaerobic form of mitochondria called hydrogenosomes that has undergone significant reductive evolution in functions and proteome [19]. Hydrogenosomes lack tricarboxylic acid cycle, respiratory complexes, and oxidative phosphorylation. In mitochondria, protein import is driven by membrane potential (Δψ) across the inner membrane and ATP hydrolysis. However, the absence of respiratory complexes in hydrogenosomes has led to the depletion of Δψ. Consequently, protein import relies mainly on ATP [20], although hydrogenosomes are bioenergetically inefficient producing only one ATP per molecule of pyruvate/malate [21]. Perhaps, these factors have led to the divergence of both targeting signals and protein import machinery in *T. vaginalis* [19, 22–24]. Except for the pore-forming Tom40, the TvTOM complex is highly divergent with two lineage-specific receptors Tom36 and Tom46, a truncated Tom22, and an uncharacterized protein Homp19 [24]. The TvTOM complex has a unique skull-shaped structure, most likely because of its stable association with Sam50 [24]. Previous hydrogenosomal proteomic analyses showed the presence of five paralogues of Tim17 family named Tim17/22/23A-E, as their classification to Tim17, Tim22, and Tim23 subfamilies was ambiguous due to the high divergence of protein sequences [10, 16, 22]. Further, homologs of Tim44, Hsp70, Mge1, Pam18, Pam16, and Tim9-10 were found in the proteome, while the rest of the known TIM subunits seem to be absent [22, 25]. Currently, it is not known how preproteins are recognized and transported across the hydrogenosomal inner membrane.

In this study, we investigate the function and structure of the TIM complex in *T. vaginalis*. Our in-depth phylogenetic, interactome, and functional analyses show that *T. vaginalis* has retained all three classes of Tim17 family proteins—Tim17, Tim22, and Tim23, which interact with each other to form a hybrid TIM22-TIM23 complex.

## Results

### Subfamily sorting of Tim17/22/23 family proteins

**Fig. 1:**
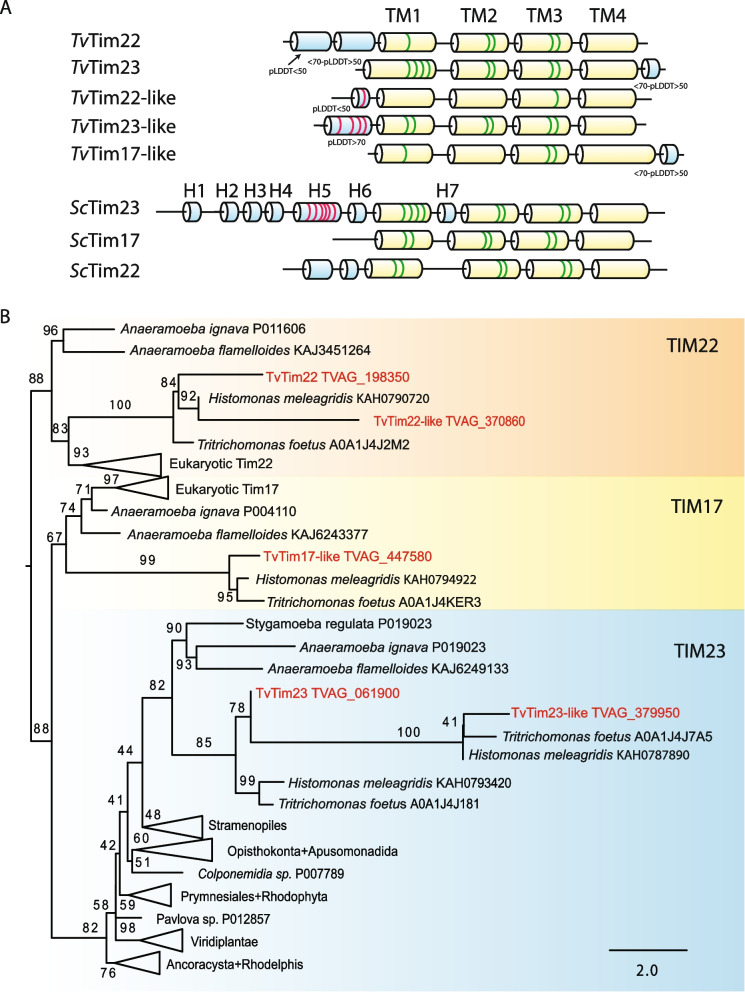
Domain analysis and phylogeny of TvTim17 family proteins. **A** Scheme of structural domains for *Trichomonas vaginalis* and *Saccharomyces cerevisiae* Tim17 family proteins. Alpha helices of *T. vaginalis* TvTim17/22/23 were predicted by AlphaFold. All transmembrane helices were predicted with high confidence (pLDDT >70), pLDDT values for other helices are indicated. Domain structure of yeast Tim23, Tim22, and Tim17 is according to [11, 13, 26–28]. Yellow: transmembrane α-helix (TM1-4); Blue: α-helix (H1-7, numbering according to Tim23 [26]; Green: glycine residues of GxxxG motif [28]; Red: negatively charged residues in H5 [26]. **B** Phylogenetic analysis of TvTim17 family proteins. The maximum likelihood (ML) tree was constructed using IQ-TREE (Best fit; LG+C50+G4 model with 91 sequences and 100 sites). Ultra-fast bootstrap values were calculated using 10,000 replicates

Comparison of predicted domain structures of *T. vaginalis* Tim17/22/23A-E with model *Saccharomyces cerevisiae* orthologs revealed that all *T. vaginalis* proteins have the propensity to form four transmembrane helixes (TM1-4) with GxxxG (G – glycine, x - any) motifs, the characteristic features of Tim17 family proteins (Fig. 1A, Additional file 1: Fig. S1). The overall domain structure of Tim17/22/23A resembles yeast Tim22 with two predicted N-terminal helixes and GxxxG motifs in TM1-3, although only a single glycine was conserved in TM1. Tim17/22/23B carries some features of yeast Tim23, including a tandem GxxxG in TM1, and conserved residues L138 and N139 between TM1 and TM2 that were implicated in interaction with Tim44. However, it lacks N-terminal IMS-facing α-helices (H1-H6), including H5 of yeast Tim23 that can bind to presequence [26]. A single N-terminal α-helix with four glutamates was predicted with high confidence only in Tim17/22/23D; however, this protein contains only a single GxxxG tandem motif at TM1. The structure of other paralogs did not reveal any significant hint of their subfamily sorting.

To perform phylogenetic analysis of Tim17/22/23 proteins, we took advantage of the recently described Anaeramoeba species, free-living relatives of Parabasalia that were reported to possess a standard set of Tim17 family proteins [29], and we also included other parabasalid species for which genome sequences are now available. The analysis provided further support for Tim17/22/23A and Tim17/22/23B being divergent orthologs of Tim22 and Tim23, respectively (Fig. 1B., Additional file 1: Fig. S2). Further, Tim17/22/23C and Tim17/22/23D also appeared within Tim22 and Tim23 subtrees respectively, although they formed longer branches. Tim17/22/23E clustered within the Tim17 subfamily, however, with lower statistical support (non-parametric bootstrap value 67, Fig. 1B, Additional file 1: Fig. S2). Furthermore, we reconstructed a phylogenetic tree only for Parabasalia species, in which the proteins clustered into three well-supported groups (Additional file 1: Fig. S3). Tim17/22/23C clustered together with Tim17/22/23A in the Tim22 group, and Tim17/22/23D with Tim17/22/23B in the Tim23 group. Tim17/22/23E appeared to have close orthologs in most parabasalid species that formed the third well-supported group (non-parametric bootstrap value 89; Additional file 1: Fig. S3). To classify Tim17/22/23A-E, we further considered the results of protein homology and structural searches summarized in Additional file 2: Table S1.

Based on these results, we concluded that Tim17/22/23A and B are orthologs of Tim22 and Tim23, respectively (hereafter TvTim22 and TvTim23, respectively), Tim17/22/23C and D are divergent Tim23 and Tim22, respectively (hereafter TvTim23-like, and TvTim22-like respectively), and Tim17/22/23E is either highly divergent Tim17 or parabasalid specific Tim17 family protein (hereafter TvTim17-like).

### The structure of TvTIM/TvTim22 complex

**Fig. 2:**
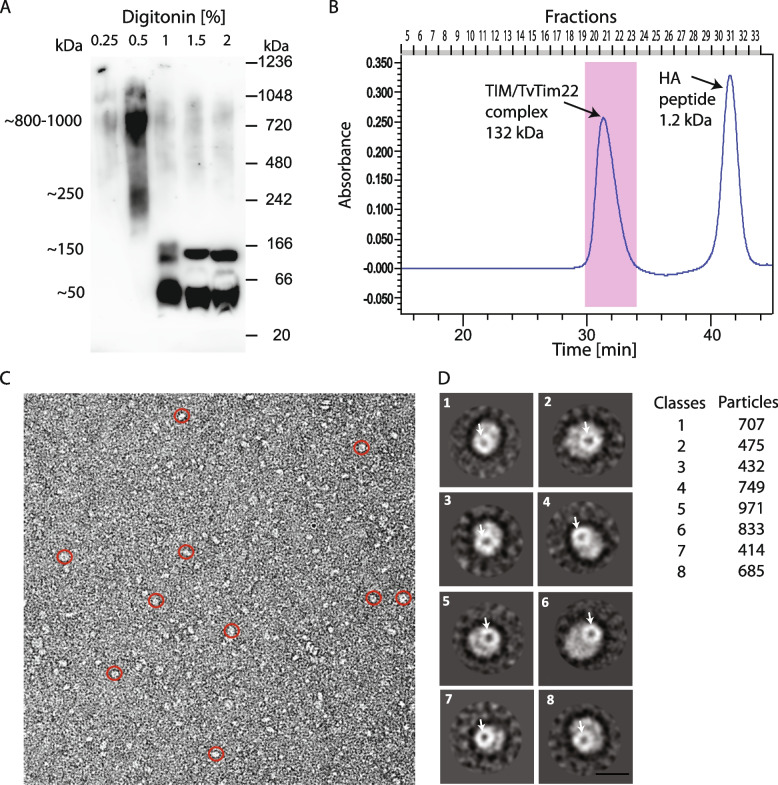
Characterization of the TvTIM/TvTim22 complex. **A** Western blot analysis of BN-PAGE for separated protein complexes. The hydrogenosomes were isolated from *T. vaginalis* cells expressing HA-tagged TvTim22, and membrane complexes solubilized by digitonin. Mouse monoclonal α-HA antibody was used to detect TvTim22. **B** Molecular weight determination of immunoprecipitated TvTIM/TvTim22 complex by size exclusion chromatography. TvTIM/TvTim22 complex was isolated from digitonin-solubilized hydrogenosomes using Dynabeads coupled with α-HA antibody. The TIM complex was eluted using HA peptides. Fractions 19–23 (highlighted in pink) were pooled and subjected to SDS-PAGE. **C** Electron microscopy analysis of single-particle TvTIM/TvTim22 complex. Overview; red circles indicate TvTIM/TvTim22 particles. **D** Eight classes represent about 10% of the TvTIM/TvTim22 particles. The resolution range is 15–18 Å. An arrow indicates a ring of the small Tims sub-complex [11, 13]. Scale bar 10 nm

Identification of TvTim22 suggested that *T. vaginalis* hydrogenosomes may contain the TIM22 complex. To elucidate this question, we first tested whether TvTim22 is present in a high molecular weight complex. The hemagglutinin-tagged (HA) TvTim22 was expressed in *T. vaginalis*, hydrogenosomes were isolated, solubilized using digitonin, and the protein complexes separated using blue native-polyacrylamide gel electrophoresis (BN-PAGE) and immunoblotted. At low digitonin concentrations (0.25–0.5%), TvTim22 migrated in high molecular weight complexes of ~800–1100 kDa, while at higher concentrations (>1%) was in smaller complexes of about 50 and 150 kDa (Fig. 2A).

Human and yeast TIM22 complex is known to be associated with small Tim chaperons, which form a twin ring [13, 30]. Since multiple small Tim paralogs were identified in the hydrogenosomal proteomes [22], we decided to isolate and visualize the protein complex containing TvTim22 (TvTIM/TvTim22). To this end, we performed coIP using Tim22-HA from isolated hydrogenosomes under native conditions. Size exclusion chromatography of the complex revealed a molecular size of 132 kDa, which is in the range of the smaller complex separated on BN-PAGE and likely corresponds to the core complex (TvTIM/TvTim22)(Fig. 2B). The purified complex was separated by SDS-PAGE (Additional file 1: Fig. S4) and analyzed by quantitative mass spectrometry (MS) that confirmed a dominant presence of TvTim22 (Additional file 3: Table S2). The structure of the purified TvTIM/TvTim22 complex was investigated by single-particle electron microscopy using negative staining and cryo-electron microscopy (cryo-EM). Unprocessed electron micrographs from negative staining mainly showed particles with one stain-filled center (representative electron micrograph in Fig. 2C). Altogether, 103,800 identified particles were subjected to multiple rounds of 2D classification in Relion 2.1, which resulted in a final dataset of 4722 particles. The 2D class averages revealed particles of size between 65 and 144 nm^2^ with a resolution between 15 and 18 Å (Fig. 2D, Additional file 4: Table S3). The particles showed a single ring-shaped structure sitting on top measuring 7–8 nm in diameter and 33–47 nm^2^ in area, and with a pore diameter of 3–5.4 nm. The position of the ring was mostly asymmetric to the surrounding mass of the complex (Fig. 2D). The mass adjacent to the small Tims ranged between 28 and 106 nm^2^ in size (Fig. 2D, Additional file 4: Table S3). Unfortunately, the cryo-EM dataset had a limited number of particles due to their tendency to adhere to the carbon film or the edges of the carbon film holes. As a result, the datasets were not appropriate for high-throughput imaging and single-particle analysis (Additional file 1: Fig. S5).

### Interactome of the TvTIM complex

**Fig. 3:**
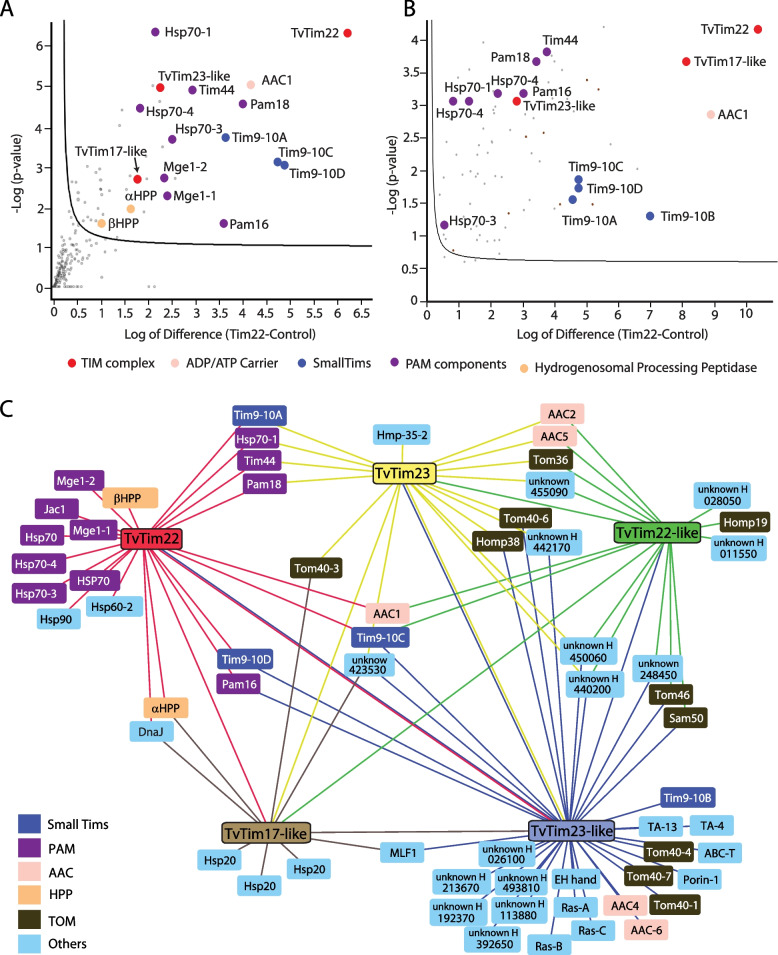
Interactome of TvTim17 family proteins. **A** Half volcano plot shows significantly enriched proteins that coIPed with TvTim22 as bait with crosslinker and **B** under native conditions without crosslinker. **C** Interactome reconstruction. Significantly enriched proteins that coIPed with each TvTim17 family protein (baits, crosslinking) were used for interactome construction. The baits are color-coded to indicate coIPed proteins. Numbers at unknown proteins correspond to TrichoDB TVAG numbers

Since *T. vaginalis* has TvTim22, TvTim23, and TvTim17-like, and TvTim22 was found to be in complex with TvTim17-like, small Tims, Tim44, and mtHsp70, we investigated whether *T. vaginalis* has a general TIM complex or both TIM22 and TIM23 complexes in detail. Hence, we reconstructed the TvTIM complex interactome by performing affinity purifications coupled with quantitative proteomics. While coIP with specific antibodies targeting the bait protein would be an ideal approach, the existence of multiple cross-reacting paralogs precludes the use of this strategy. Thus, all TvTim17 family paralogs were expressed with HA-tag in trichomonads and were pulled down along with interacting proteins from isolated hydrogenosomes after chemical crosslinking using an anti-tag antibody. The hydrogenosomes from the non-transformed parent strain were used as a negative control, which was processed identically as hydrogenosomes from transformed cells to distinguish specific interactions with proteins of interest and non-specific background. The eluted samples were subjected to label-free quantitative mass spectrometry. From the dataset of statistically enriched proteins (FDR=0.05, S=1; relative to the negative control), we filtered out all proteins that were identified in only one of the replicates out of three with functions unrelated to protein import and proteins of unknown function except those, which coIP with at least two baits, and we also included all unknown proteins that were previously found in the hydrogenosomal proteome (Fig. 3, Additional file 1: Fig. S6, Additional file 5: Table S4A-E). CoIP of TvTim22 confirmed that TvTIM/TvTim22 includes TvTim17-like protein, small Tims, PAM components, and AAC1 (Fig. 3A). TvTim22 coIP experiment was repeated under native conditions without chemical crosslinking to limit possible transient interactions of the translocase with imported substrates (Fig. 3B). However, no significant difference was observed, which indicates that the interactions between TvTim22 and other partner proteins are rather stable. Interestingly, several PAM components were shared between TvTim22 and TvTim23 (Tim44, Pam18, and Hsp70-1), as well as TvTim22 and TvTim23-like (Pam16) (Fig. 3C). Additionally, TvTim22 co-eluted both α and β subunits of hydrogenosomal processing peptidase (HPP). Both TvTim22 and TvTim23 could pull down TvTim23-like and TvTim23-like could reciprocally pull down both TvTim22 and TvTim23 (Fig. 3C). TvTim17-like was linked with all TvTim17 family proteins suggesting its more general function. Importantly, four small Tims (Tim9-10A-D) were shared by various combinations of TvTim22/TvTim22-like and TvTim23/TvTim23-like proteins, but none of them was pulled down by TvTim17-like protein. Interestingly, all TvTim17 family proteins except TvTim22 pulled down several components of the TOM-SAM complex (five paralogs of Tom40, receptors Tom36, and Tom46, Homp19, and Sam50).

Of note, TvTim22-type proteins could pull down TvTim17- and TvTim23-type proteins, and vice versa which suggests that the TvTIM complex has a hybrid TIM22-TIM23 character, and possibly includes all three members of the TvTim17 protein family: TvTim17-like, TvTim22/22-like, and TvTim23/23-like in various combinations of paralogs, small Tim9-10 chaperones, and interacts with PAM and possibly TOM-SAM complex.

### TvTim22 interacts with Tim44 and AAC1

**Fig. 4:**
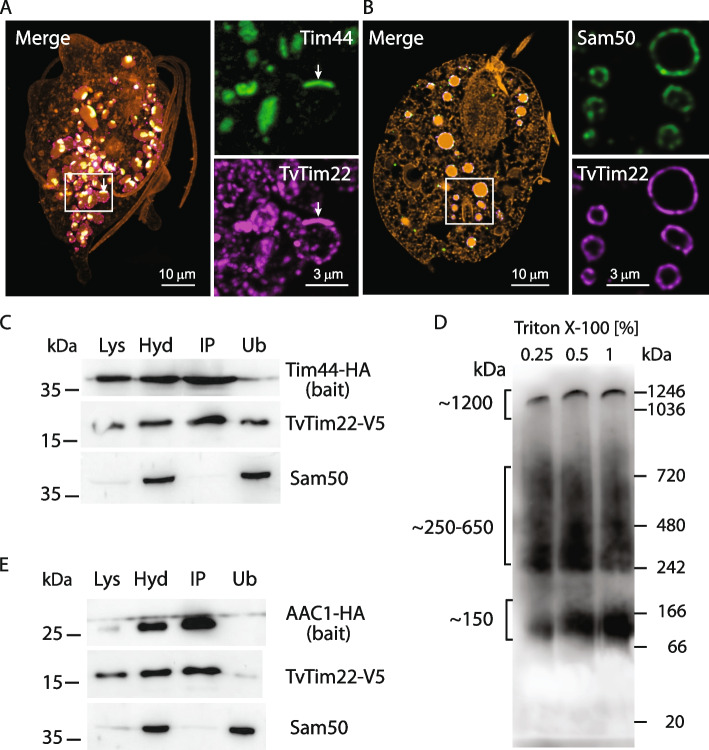
TvTim22 interacts with Tim44 and AAC1. **A** Expansion immunofluorescence microscopy of *T. vaginalis* expressing HA-tagged TvTim22 (in red) and V5-tagged Tim44 (in green). The cell was visualized using N-hydroxysuccinimidyl(NHS)-ester (brown). White arrows indicate Tim44 and TvTim22 patches that are associated with the hydrogenosomal membrane. **B** Unlike Tim44, Sam50, a control outer membrane protein, was evenly distributed. **C** Tim44 (bait) co-immunoprecipitated TvTim22. Western blot analysis of cell lysate (Lys), isolated hydrogenosomes (Hyd), immunoprecipitated fraction (IP), and unbound fraction (Ub). For IP, 1% Triton X-100 was used to solubilize hydrogenosomes at a protein concentration of 1 mg/ml. **D** Western blot analysis of BN-PAGE for separated protein complexes. The hydrogenosomes were isolated from *T. vaginalis* cells expressing HA-tagged Tim44 and membrane complexes were solubilized by 1% Triton X-100. Mouse monoclonal α-HA antibody was used to detect Tim44-containing protein complexes. **E** AAC1 (bait) co-immunoprecipitated TvTim22. Western blot analysis as in **C**

In support of the TIM-PAM interaction, we selected Tim44 for further investigation. HA-tagged Tim44 and V5-tagged TvTim22 were co-expressed in *T. vaginalis* and their localization in hydrogenosomes was confirmed by immunofluorescence microscopy and subcellular fractionation (Fig. 4, Additional file1: Fig. S7). To obtain higher spatial resolution for both TvTim22 and Tim44 in hydrogenosomes, we adopted expansion microscopy for *T. vaginalis*. This method displayed TvTim22 as a ring of labeled hydrogenosomal membranes. The ring was formed by discrete clusters and often a single more prominent patch. Tim44 was associated with the membrane from the matrix side predominantly in the area of TvTim22 patches, while only very weak labeling was seen in other membrane areas (Additional file 1: Fig. S7). As the protein localization might be affected by the gene expression under the control of a strong promotor in the TagVag vector derived from the hydrogenosomal succinyl CoA synthetase (SCS) (Rada et al., 2015), Tim44 was expressed also under the control of native promotor. Based on transcriptomic data, the native promoter was estimated to be approximately two orders of magnitude less efficient than the SCS promotor (Gould et al., 2013). However, despite the difference in promoter strength, the localization pattern of Tim44 was found to be consistent (Fig. 4A, Additional file1: Fig. S7). Distribution of HA-tagged Sam50 which was used as a control outer membrane protein unrelated to TvTIM complex revealed a different pattern when compared with Tim44 (Fig. 4B).

Next, the TvTim22-Tim44 interaction was tested by reciprocal coIP experiment. Tim44-HA could pull down TvTim22-V5, while Sam50 was not eluted with Tim44 (Fig. 4C, Additional file1: Figure S8). As TvTim22 was present in high molecular complexes (Fig. 2A), we expected a similar behavior for Tim44. Indeed, BN-PAGE of solubilized hydrogenosomal protein complexes and immunoblotting detected HA-tagged Tim44 in a complex of ~150 kDa, and also in complexes of higher molecular weight of about 250–650 (Fig. 4D).

The purification of the TvTIM complex under native conditions and TIM complex interactome analysis also revealed that TvTim22/22-like and TvTim23/23-like pulled down several paralogs of AAC suggesting that AAC might be an integral component of the TvTIM complex (Fig. 3A–C). To this end, a strain co-expressing AAC1-HA and TvTim22-V5 was prepared. The coIP experiment confirmed that AAC-HA reciprocally pulled down TvTim22-V5, which further supports the possibility that AAC1 and TvTim22 are constituents of a common complex (Fig. 4E, Additional file 1: Fig. S8).

### The TvTIM/TvTim22 complex facilitates the import of proteins into the hydrogenosomal matrix

**Fig. 5:**
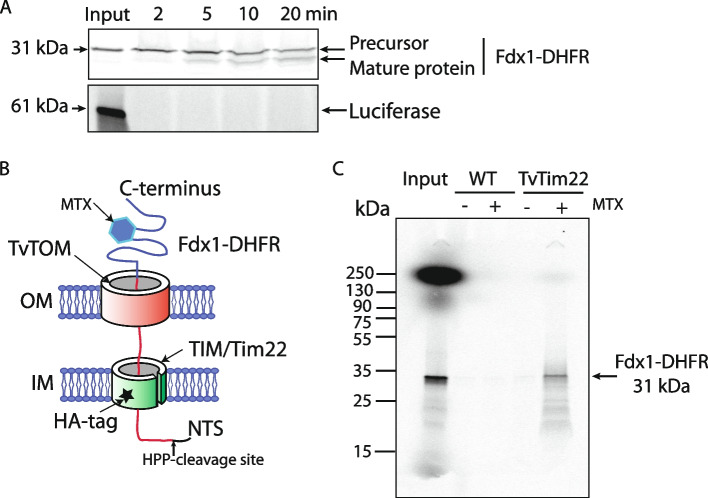
TIM/TvTim22 complex is involved in the import of NTS-carrying matrix proteins. **A** Time-course of radiolabeled Fdx1- DHFR import into isolated hydrogenosomes in vitro. Luciferase was used as a negative control. **B** A schematic depiction of Fdx1-DHFR arrested in hydrogenosomal translocases upon the addition of methotrexate (MTX). **C** Autoradiography of the arrested Fdx1-DHFR that was coIPed following crosslinking with DSP when HA-tagged TvTim22 was used as bait; Input (100% of ^35^S-Fdx1-DHFR), Eluate (100%). 1 % Triton X-100 was used to lyse the organelles

To investigate the function of the hydrogenosomal TvTIM/TvTim22 complex, we performed in vitro protein import and coIP assays. As substrates, we used NTS-containing matrix protein ferredoxin-1 (Fdx1), and inner membrane protein AAC1 that were fused to dihydrofolate reductase (DHFR) at the C-terminus. Luciferase was a negative control. The substrates were synthesized in vitro in the presence of [^35^S]-methionine. The autoradiograph showed a time-dependent import of Fdx1-DHFR into isolated hydrogenosomes, and the appearance of mature Fdx1-DHFR that requires the cleavage of the NTS by HPP in the matrix (Fig. 5A, Additional file 1: Fig. S9). All the samples were treated with proteinase K, which confirmed that Fdx1-DHFR was membrane-protected and thus indeed imported into hydrogenosomes (Fig. 5A). Methotrexate induces the folding of DHFR and therefore arrests the translocating protein at the protein import site (Fig. 5B). Thus, to prove that the TvTIM/TvTim22 complex is involved in the substrate import, we performed an in vitro import assay with Fdx1-DHFR in the presence or absence of methotrexate using TvTim22-HA containing hydrogenosomes. After the import reaction, the proteins were crosslinked, the hydrogenosomes were solubilized, and TvTim22-HA was pulled down. Hydrogenosomes from non-transfected parent strain were used as a negative control. Autoradiography of the eluted samples revealed the presence of arrested Fdx1-DHFR associated with the TvTIM/TvTim22 complex when methotrexate was added (Fig. 5C). When AAC1-DHFR was used as a substrate, we could import the radiolabeled protein into hydrogenosomes under the same experimental conditions, however, in the presence of methotrexate, the arrested AAC1-DHFR remained insoluble in the hydrogenosomes (Additional file 1: Fig. S10). Taken together, these results demonstrate that the hydrogenosomal TvTIM/TvTim22 complex is involved in the import of proteins into the hydrogenosomal matrix, although it remains to be established, which component forms the protein translocation channel. Further investigations are required to resolve the import of inner membrane proteins.

## Discussion

Protein import is a fundamental process for the biogenesis and function of mitochondria in eukaryotic cells. However, little is known about this process in mitochondria-derived organelles such as hydrogenosomes. In this work, we investigated the structure and function of the TIM complex in the inner hydrogenosomal membrane of a human parasite *T. vaginalis*. While most eukaryotes employ specialized translocases in the mitochondrial inner membrane—TIM23 and TIM22 complexes for the import of presequence-containing matrix, and MCPs, respectively, our data indicate that *T. vaginalis* seems to contain a hybrid TvTIM complex, most likely composed of various combinations of divergent forms of all three TvTim17 family proteins—TvTim22/TvTim22-like, TvTim23/TvTim23-like, and TvTim17-like. The TvTIM complex was found to be tightly associated with small Tim chaperones towards the IMS, AACs in the inner membrane, and interacted with PAM towards the matrix. Our experiments have demonstrated that the import of matrix proteins is mediated by the TvTIM complex that includes TvTim22. The structure of the TvTIM/TvTim22 complex was visualized by single-particle microscopy revealing the presence of a single ring that was most likely formed by small Tims.

The high diversity of TvTim17 family proteins in anaerobic protists is noteworthy. In *T. vaginalis*, five hydrogenosomal paralogs were identified by proteomics previously; however, their classification was not clear [10, 16, 22]. Our reciprocal homology searches, domain structure, and phylogenetic analyses assigned two hydrogenosomal proteins to TvTim22 and TvTim23 groups respectively with high confidence. Furthermore, phylogeny suggested that the other three homologs are highly divergent members of Tim22, Tim23, and Tim17 subfamilies, although with low support for TvTim17-like protein. The identification of Tim17 family proteins is even more challenging in anaerobes possessing the most reduced form of mitochondria named mitosomes. In *Giardia intestinalis*, the protein with limited homology to Tim17 was identified using a metamonad-specific hidden Markov model [16]. Both in *Entamoeba histolytica*, which contains mitosomes, and its free-living relative *Mastigamoeba balamuthi*, which contains hydrogenosomes, no TIM component has been identified so far [31, 32]. The observed diversity is most likely a result of the functional adaptation of the TIM complex to import proteins across the inner hydrogenosomal/mitosomal membrane under anaerobiosis. In mitochondria, two major factors are required for the protein import, the inner membrane Δψ and ATP hydrolysis. In yeast, Δψ which is negative on the matrix side energizes the TIM23 complex and causes an electrophoretic effect on the translocation of proteins with positively charged NTSs across the channel [33–35]. Additionally, the integration of carrier proteins into the lipid bilayer by the TIM22 complex is strictly dependent on Δψ [30]. As a result of very low Δψ, *T. vaginalis* NTSs are shorter with lower positive net charge [36, 37]. Additionally, *T. vaginalis* does not have typical presequence-binding receptors such as Tom20 and Tim50 [22, 24]. Moreover, NTSs are not essential for import but rather contribute to the import efficiency, and the hydrogenosomal protein translocation can proceed entirely without NTS [22, 23, 38]. The limited role of Δψ supports the observation that hydrogenosomal protein import was not abolished with the protonophore m-chlorophenyl-hydrazone (CCCP) at concentrations that dissipate Δψ in mitochondria [20, 39]. These evolutionary comparisons and experimental data indicate that loss of Δψ was the major factor that drove the diversification of Tim17 family proteins. The character of the force facilitating the movement of hydrogenosomal preproteins across TIM is still unclear. However, Δψ-independent protein import is not limited to hydrogenosomes. The ability of mitochondrial TIM23 complex to import certain proteins only upon Δψ dissipation has been reported [40].

In contrast to Δψ, the hydrogenosomal protein import remains dependent on ATP [20, 41]. Accordingly, complete ATP-dependent PAM machinery is conserved. The role of PAM machinery as the translocase motor in hydrogenosomes supports our coIP data indicating the association of *T. vaginalis* TIM complex with all the five PAM components: scaffold protein Tim44, ATP-driven chaperone mtHsp70, co-chaperones Pam16 and Pam18, and nucleotide-exchange factor Mge1. In addition, after lysis of mitochondrial membranes, both Tim44 and TvTim22 co-migrated in the complex of about 132 kDa and were present in the purified TvTIM complex. In yeast mitochondria, Tim44 was shown to transiently interact with Tim17 and/or Tim23 [42–44]. Isolated TIM23 complex contained Tim17-Tim23-Tim44 heterotrimer, with varied non-core subunits. Of note, mitochondrial Tim44 dissociated during the complex purification and required stabilization by a Fab fragment of the antibody [6, 45]. Furthermore, Tim23 and Tim44 formed distinct clusters in the human mitochondria when directly visualized by superresolution microscopy [46]. The association of Tim44 with *T. vaginalis* TIM complex appeared to be more stable. Tim44 did not dissociate during the purification of the 132-kDa TIM complex and could migrate at high molecular weight complex on BN-PAGE. Interestingly, expansion immunofluorescence microscopy revealed an association of TvTim22 and Tim44 predominantly in a large single structure at the membrane. Tim44 seemed to be attached to the inner membrane from the matrix side as expected. We cannot exclude the possibility that the formation of the large cluster is an episomal expression artifact. However, the same Tim44 distribution was observed when the Tim44 gene was expressed under the control of both strong and native promotors, which limits the risk of an artifact [41].

Visualization of the TvTIM/TvTim22 complex using single-particle electron microscopy revealed particles with a single ring-shaped structure with a central pore, which is a typical structure of a small Tim hexamer. [47]. Currently, yeast and human TIM22 complexes and yeast TIM23 complex have been directly visualized by electron microscopy [11, 13, 30, 45, 48]. Unlike the TvTIM/TvTim22 complex, the yeast/human TIM22 complex forms a twin small Tims hexamer, while small Tims are not associated with the TIM23 complex. To what extent the single chaperone sub-complex in TvTIM is unique across eukaryotes is unclear as visualizations in other lineages are not available. Another difference provides a comparison of inner pore size. The small Tim inner pore size diameter is around 3.4 nm, which is similar to the pore diameter of TvTOM (2.5–3 nm). The yeast inner pore size diameter of the TOM channel is in the same range (2.5 nm). However, in yeast, the small Tim pore diameter is smaller (1.6 nm), about half of trichomonad small Tims [30]. The area adjacent to small Tims in the TvTIM/TvTim22 complex had a mass of variable size (28.4–106.1 nm), which is consistent with a set of PAM components and other proteins identified by proteomic analysis of the complex. In yeast, the isolated TIM22 complex showed a twin small Tim hexamer about 11 nm in length without adjacent mass [30], or with mass variability [13], while the TIM23 complex was associated with an extra mass of putative Tim44 and Pam16-Pam18.

The TIM22, and TIM23 complexes have one, and two translocation channels formed by Tim22 and Tim17-23, respectively [45, 48]. Based on our interactome and structural analyses, which suggest that TvTim22/22-like, TvTim23/23-like, and TvTim17-like are possibly associated in a common TvTIM complex, we may estimate that the TvTIM complex contains at least two or more channels for protein import. Interestingly, unlike TvTIM, the structure of the TvTOM complex seems to be more conserved in hydrogenosomes. Isolated TvTOM was shown to be composed of twin, and triple pores, which is similar to core, and holo complexes in fungi, respectively [24].

Of note, four hydrogenosomal TvTim17 family proteins coIP components of TvTOM, including five of seven known Tom40 paralogs, specific hydrogenosomal receptors Tom46 and Tom36, and Sam50, which forms a stable complex with TvTOM [24]. The outer and inner hydrogenosomal membranes in *T. vaginalis* are tightly apposed without apparent intermembrane space and cristae [49]. Thus, domain and proteins that cross IMS to link TIM and TOM into a dynamic supercomplex are likely dispensable. Indeed, the Tom22 *trans* domain, which faces IMS in mitochondria, Tim50 receptor, and Tim21, which are involved in the efficient handover of substrates from TOM to TIM23 are absent in *T. vaginalis*. Neither TvTim23 nor TvTim23-like hydrogenosomal proteins possess an N-terminal domain homologous to yeast Tim23 that crosses IMS and spans the mitochondrial outer membrane [50]. Similarly, proteins with a large IMS domain associated with Tim22 such as yeast Tim54 or human AGK/Tim29 were not identified in the hydrogenosomal proteome [22]. Thus, a direct association of TvTom40 and TvTim17 family proteins into a supercomplex seems to be a plausible explanation of our coIP data and an attractive hypothesis for further studies.

## Conclusions

The adaptation of *T. vaginalis* for life under anaerobic conditions has resulted in lineage-specific modifications of protein import machinery and pathways. Although the reduction of respiratory pathways and consequent loss of Δψ had a strong impact on the entire protein import machinery, the structure, and composition of TvTIM are particularly divergent and evolved to a single hybrid TIM22-TIM23 translocase. This arrangement seems to be an evolutionary unique solution, which is unprecedented in other eukaryotes.

## Materials and methods

### Cell cultivation

*T. vaginalis* strain T1 and the recombinant strains were grown in tryptone-yeast extract-maltose medium, pH 6.2 supplemented with 10% (v/v) heat-inactivated horse serum either without or with 200 µg/ml geneticin 418 respectively at 37 °C. Recombinant *Escherichia coli* strains were grown on Luria-Bertani medium with 100 µg/ml of ampicillin at 37 °C.

### Preparation of recombinant strains

The genes encoding TvTim22 (TVAG_198350), TvTim23 (TVAG_061900), TvTim22-like (TVAG_370860), TvTim23-like (TVAG_379950), TvTim17-like (TVAG_447580), Tim44 (TVAG_008790), Sam50 (TVAG_178100), and AAC1 (Hmp31) (TVAG_237680) were cloned into pTagVag vector fused to either di-Haemagglutinin (HA) tag or di-V5 tag at the C-terminus [51, 52]. To express Tim44 under the control of the native promotor, the α-succinyl CoS synthetase promotor in pTagVag vector was replaced with 292-bp sequence upstream of the Tim44 open reading frame. The plasmids were transfected by electroporation as described previously [24]. The oligonucleotides used are listed in Additional file 6: Table S5.

### Subcellular fractionation, BN-PAGE, and immunoblotting

Trichomonad cells were harvested, lysed by sonication, and the subcellular fractions were prepared by differential centrifugation as described previously [53]. BN-PAGE was performed with isolated hydrogenosomes as described previously using either varying concentrations of digitonin (0.25–2.5%) or triton X-100 (0.25–1%) [24]. The samples were electrophoresed, blotted, decorated with α-HA antibody, and developed using chemiluminescence.

### Standard and expansion immunofluorescence microscopy

Confocal immunofluorescence microscopy was performed as previously described [41]. Recombinant proteins were detected using mouse monoclonal α-HA (ExBio s.r.o., Vestec, Czech Republic) and rabbit α-V5 (Abcam, Cambridge, UK) antibodies and visualized with Alexa Fluor 488 donkey α-mouse and Alexa Fluor 594 donkey α-rabbit antibodies (Thermo Fisher Scientific). The slides were mounted using Vectashield containing DAPI (4′, 6-diamidino-2-phenylindole) (Vector laboratories). The cells were observed using a Leica TCS SP8 DM6 CFS confocal microscope, the images were deconvolved with Huygens Professional version 19.10 SVI software, and further processed using Fiji [54].

Expansion microscopy was adapted for *T. vaginalis* based on the described method [55]. Recombinant TvTim22 and Tim44 were detected using mouse α-HA (ExBio, Prague, Czech Republic) and rabbit monoclonal α-V5 (Abcam, Cambridge, UK) antibodies, respectively, and visualized with Alexa Fluor 488 donkey α-mouse and Alexa Fluor 647 donkey α-rabbit antibodies (Thermo Fisher Scientific, Waltham, USA). Amine-reactive reagent N-hydroxysuccinimidyl(NHS)-ester ATTO 550 (ATTO-TEC, Siegen, Germany) was used for the overall cell structure visualization (1 µg/ml in PBS for 2 h). The cells were observed using a spinning disk confocal microscope Nikon CSU-W1, and the images were processed with Huygens Professional version 19.10 (Scientific Volume Imaging) and the Imaris 9.7.2 Package for Cell Biologists (Bitplane AG, Zurich, Switzerland).

### Co-immunoprecipitations

Hydrogenosomes were isolated from various Trichomonas strains expressing HA-tagged proteins (TvTim22, TvTim23, TvTim22-like, TvTim23-like, TvTim17-like, Tim44, and AAC1) and the wild-type strain (a negative control). Protein crosslinking experiments were performed for isolated hydrogenosomes using DSP (Dithiobis(succinimidyl propionate), Thermo Scientific) as described previously [24]. Briefly, hydrogenosomes in isolation buffer (IB) containing 225 mM sucrose, 10 mM KH_2_PO_4_, 20 mM HEPES, 0.5 mM KCl, 5 mM MgCl_2_, and 1 mM EDTA [pH 7.2], and supplemented with protease inhibitors, were crosslinked in the isolation buffer containing 1 mM DSP for 30 min at 25 °C. The crosslinker was quenched with 50 mM Tris, pH 7.5 [24]. For the native coIP of TvTim22, the crosslinker was omitted. Next, hydrogenosomes were twice washed in IB and lysed in TSG buffer (50 mM Tris/HCl, 150 mM NaCl, 10% glycerol, EDTA-free cOmplete protease inhibitor cocktail (Roche), pH 7.2) containing 1% digitonin or 1% Triton X-100. The clarified extract obtained by centrifugation was then incubated with Dynabeads (Thermo Fisher Scientific) coupled with α-HA antibody. The beads were five times washed in TSG buffer containing 0.1% detergent. Further, samples for immunoblotting were eluted with SDS-PAGE sample buffer and analyzed using α-HA, α-V5, and α-Sam50 antibodies, or the beads containing protein samples were subjected to LFQ-MS along with the wild-type strain as a control as described previously [24]. The MS data were obtained from 3 independent experiments for each immunoprecipitated protein. The coupling of the α-HA antibody to the Dynabeads was performed according to the manufacturer’s instructions.

### TIM complex purification, size exclusion chromatography, and mass spectrometry

Trichomonas cells expressing HA-tagged TvTim22 were harvested and hydrogenosomes were isolated. Isolated hydrogenosomes were lysed using TSG buffer containing 1% digitonin (Merck Millipore) or 1% triton X-100. The clarified extract obtained by centrifugation was then incubated with Dynabeads (Thermo Fisher Scientific) coupled with α-HA antibody. The beads were five times washed in TSG buffer containing 0.1% detergent, and the HA-tagged protein was eluted using TSG buffer containing 1 mg/ml HA peptide (Thermo Fisher Scientific), 0.2% digitonin or 0.05% triton X-100 and protease inhibitors. The coupling of the α-HA antibody to the Dynabeads was performed according to the manufacturer’s instructions. For size exclusion chromatography, the eluted sample was applied to Superose 6 10/300 GL (GE Healthcare) equilibrated with running buffer (50 mM Tris/HCl, 150 mM NaCl, 5% glycerol, and 0.05% triton X-100) coupled with chromatography system BioLogic Duoflow (BioRad). Peak fractions were pooled and subjected to SDS-PAGE and silver staining. Bands on the gel were sliced and proteins were identified by mass spectrometry.

### Mass spectrometry

Individual bands containing proteins were destained using ProteoSilver Plus – Silver Stain Kit (Sigma Aldrich) according to manufacturer instructions. After destaining, gels were dried in acetonitrile (ACN). Disulfide bonds were reduced using 10 mM dithiothreitol, free cysteine residues were blocked using 55 mM iodoacetamide, and proteins were digested with trypsin. After digestion, 150 µl of 50% ACN with 0.5% formic acid was added and submitted to two rounds of 30 min sonication. The peptides were dried, reconstituted in 2% ACN with 0.1% trifluoroacetic acid, and injected into Ultimate 3000 Nano liquid chromatography coupled to Thermo Orbitrap Fusion (Thermo Scientific)(nanoLC-MS).

Label-free quantitative mass spectrometry (LFQ-MS) analysis was performed as described previously [52]. Briefly, the samples from coIP experiments were digested with trypsin, and the peptides were subjected to nanoLC-MS. The MS/MS spectra were searched against the *T. vaginalis* database (Trichomonas Genome Resource, www.trichdb.org, containing 59,862 entries), the quantifications were performed with the label-free algorithms, and the data analysis was performed with Perseus 1.6.10.43 software. The false discovery rate (FDR) was set to 5%, parameter S=1. The MS data were obtained from four independent coIP experiments for each immunoprecipitated protein. The data have been deposited to the ProteomeXchange consortium via the PRIDE [56] partner repository with the data set identifier PXD047649.

### Transmission electron microscopy

Five microliters of purified TIM complex were applied to copper electron microscopy grids (EMS200-Cu) covered with 20-nm carbon films, which were glow discharged for 40 s with a 5 mA current prior to specimen application. The excess sample was removed after 1 min by blotting (Whatmann no. 1 filter paper) for 1 to 2 s, and the grid was immediately stained with 5 μL of 1% Uranyless EM stain for 1 min 20 s, and blotted to remove the excess stain for 1 to 2 s. A dataset was acquired with a Tecnai F20 microscope (Thermo Fisher Scientific) operating at an accelerating voltage of 200 kV, with a FEI Eagle 4K CCD camera, at a magnification of ×78,000 with a final pixel size of 1.38 Å. Micrographs were acquired with defocus ranging from 2 to 5 μm. After quality inspection and determination of Contrast Transfer Function (CTF) parameters with the GCTF program [ref gctf], 1042 micrographs were subjected to particle picking. Approximately 103,800 particles were picked using semi-automated mode from e2boxer.py routine of the EMAN2 program [57]. The particles were extracted with a box size set to 180 pixels. The particle set was subjected to multiple rounds of 2D class averaging in Relion 2.1 [58, 59] to clean the data set. All 2D classifications comprised 25 iterations. This procedure resulted in a set of representative class averages. For each selected/representative class, the size of the particle was measured and calculated using Fiji [54].

### In vitro protein import assay

Radiolabeled ferredoxin 1 (TVAG_003900) and AAC1 (TVAG_237680) fused at C-terminus with DHFR and luciferase were synthesized in vitro in the presence of L-[^35^S] methionine (MGP s.r.o. Zlín, Czech Republic) according to the manufacturer’s instructions (PURExpress™ In Vitro Protein Synthesis Kit, New England BioLab, Inc., Ipswich, USA). Standard in vitro protein import time-course experiments, and in vitro protein import-arrest and crosslinking coIP assays were performed using isolated hydrogenosomes carrying HA-tagged TvTim22 and radiolabeled precursors as described previously except for 1% Triton X-100 was used to solubilize instead of 0.5% [24]. The samples were electrophoresed, and the gel was vacuum-dried. The gel was exposed for 4–5 days prior to phosphorimaging using Typhoon TLA 7000 scanner (GE Healthcare Europe GmbH, Freiburg, Germany).

### Bioinformatic analyses

Amino acid sequences of *T. vaginalis* Tim17/22/23A-D and Tim17-like [22] were retrieved from TrichoDB (https://trichdb.org/trichdb/app) and used as queries for homology searches in genomes and transcriptomes at the National Center for Biotechnology and Information (NCBI). The assembled transcriptome and protein dataset for *A. flamelloides* and *A. ignava* was acquired from the FigShare website 10.6084/m9.figshare.12205517.v1 [29].

Multiple sequence alignment was performed using MAFFT [60], and the alignment was trimmed with BMGE [61]. The maximum likelihood (ML) tree was constructed with IQ-TREE v.1.6.7 [62] using the LG+C50+G4 model. Ultra-fast bootstrap values were calculated using 10,000 replicates.

The domain structure predictions of Tim17 family proteins were based on AlphaFold [63] and TMHMM2.0 (Department of Health Technology, Denmark).

### Supplementary Information

**Additional file 1:** **Fig S1.** Protein sequence alignment of TvTim17 family proteins with Saccharomyces cerevisiae Tim17, Tim22, and Tim23. **Fig S2.** Phylogenetic analysis of Tim17 family proteins. **Fig S3.** Phylogenetic analysis of Tim17 family proteins in parabasalids. **Fig S4.** Analysis of immunoprecipitated TIM/TvTim22 complex. **Fig S5.** Unprocessed cryo-electron microscopy of single-particle TvTIM/TvTim22 complex. **Fig S6.** Heat map of protein quantity (LFQ values) coIPed with TvTim22, TvTim22-like, TvTim23, TvTim23-like, TvTim17-like as baits using crosslinker, or without crosslinker (TvTim22 Native). **Fig S7.** Cell localization of TvTim22 and Tim44. **Fig S8.** Full immunoblots presented in Figures 4C and E. **Fig S9.** Full autoradiograms presented in Figure 5. **Fig S10.** In vitro import of AAC1 into *T. vaginalis* hydrogenosomes.

**Additional file 2:Table S1.** Summary of phylogenetic inferences, homology, and structural predictions.

**Additional file 3:Table S2.** Identification of proteins co-purified with TvTim22 using mass spectrometry. TvTim22 was utilized as bait and coIP was performed without protein-crosslinking.

**Additional file 4:Table S3.** Parameters of representative classes of TIM/TvTim22 particles selected from a dataset of electron microscopy images.

**Additional file 5:Table S4**A-E. Mass spectrometry identification of proteins that co-immunoprecipitated with *T. vaginalis* Tim17 family proteins. CoIP was performed with protein-crosslinking.

**Additional file 6:Table S5.** A list of primers.

## Data Availability

The mass spectrometry proteomics data have been deposited to the ProteomeXchange Consortium via the PRIDE [64] partner repository with the dataset identifier PXD047649.

## Abbreviations

PAM: Presequence translocase-associated motor
AAC: ADP/ATP carrier
BN-PAGE: Blue native-polyacrylamide gel electrophoresis
CCCP: Carbonyl cyanide m-chlorophenyl-hydrazone
coIP: Co-immunoprecipitation
DHFR: Dihydrofolate reductase
DSP: Dithiobis(succinimidyl propionate)
FDR: False discovery rate
Fdx1: Ferredoxin-1
HA: Hemagglutinin
HPP: Hydrogenosomal processing peptidase
Δψ: Membrane potential
IMS: Intermembrane space
LECA: Last eukaryotic common ancestor
LFQ-MS: Label-free quantitative mass spectrometry
MCP: Metabolite carrier proteins
ML: Maximum likelihood
MTX: Methotrexate
NCBI: National Center for Biotechnology and Information
NTS: N-terminal sequence
TIM: Translocase of the Inner Membrane
TM: Transmembrane helices
TOM: Translocase of the Outer Membrane
TYM: Tryptone-yeast extract-maltose
