# Supplementary material for: A hybrid TIM complex mediates protein import into hydrogenosomes of Trichomonas vaginalis
Source: BMC Biol. 2024 Jun 3;22:130. doi: 10.1186/s12915-024-01928-8 (PMC11145794; doi:10.1186/s12915-024-01928-8)
Supplement: Supplementary file 1 — Additional file 1: Fig S1. Protein sequence alignment of TvTim17 family proteins with Saccharomyces cerevisiae Tim17, Tim22, and Tim23. Fig S2. Phylogenetic analysis of Tim17 family proteins. Fig S3. Phylogenetic analysis of Tim17 family proteins in parabasalids. Fig S4. Analysis of immunoprecipitated TIM/TvTim22 complex. Fig S5. Unprocessed cryo-electron microscopy of single-particle TvTIM/TvTim22 complex. Fig S6. Heat map of protein quantity (LFQ values) coIPed with TvTim22, TvTim22-like, TvTim23, TvTim23-like, TvTim17-like as baits using crosslinker, or without crosslinker (TvTim22 Native). Fig S7. Cell localization of TvTim22 and Tim44. Fig S8. Full immunoblots presented in Figures 4C and E. Fig S9. Full autoradiograms presented in Figure 5. Fig S10. In vitro import of AAC1 into T. vaginalis hydrogenosomes. [file 12915_2024_1928_MOESM1_ESM.pdf]

Additional file 1: Supplementary figures

Makki et al.: 2024, A hybrid TIM complex mediates protein import into hydrogenosomes of *Trichomonas vaginalis*

Figure S1. Protein sequence alignment of TvTim17 family proteins with *Saccharomyces cerevisiae* Tim17, Tim22, and Tim23. The structural prediction for Tim17 is according to [1], Tim23 according to [2], Tim22 according to [3,4,5]. Structures of TvTim17 family proteins were predicted with AlphaFold (https://alphafold.ebi.ac.uk/). Helices' predictions with pLDDT>70 are underlined. Cyan: Alpha helices (H1-8, numbering referred to yeast Tim23, [6]; Yellow: transmembrane domains (TM1-4). Green: GxxxG motifs; violet, negatively-charged residues (D65, D72, E74, E75, E76, and E85) at H5 of Tim23 that interact with positively charged residues at NTS [6]; Gray: aspartates (D4, D8) at Tim17 N-terminal, which were implicated in the recognition of NTSs [7]. H6, putative voltage sensor [6]; H7, Tim44-binding helix with conserved L138 and N139 [6]. Red: C42 and C141 form disulfide bond in Tim22 to stabilize TM1-TM2 [5].

```
                           H1                H2          H3          H4

TvTim22       ---------- ---------- ---------- ---------- ---------- --MIHTALEP DATTVKGLAS  18
TvTim23       ---------- ---------- ---------- ---------- ---------- ---------- ----------  1
TvTim22-like  ---------- ---------- ---------- ---------- ---------- ---------- ----MISTAD  6
TvTim23-like  ---------- ---------- ---------- ---------- ---------- ---------- --MAKALTKE  8
TvTim17-like  ---------- ---------- ---------- ---------- ---------- ---------- ----------  1
Tim17         ---------- ---------- ---------- ---------- ---------- ---------- ----------  1
Tim23         MSWLFGDKTP TDDANAAVGG QDTTKPKELS LKQSLGFEPN INNIISGPGG MHVDTARLHP LAGLDKGVEY  70
Tim22         ---------- ---------- ---------- ---------- ---------MV YTGFGLEQIS PAQKKPYNEL  22

                  H5                    H6           TM1

TvTim22       YLIPITAEEV LYRNAGENPV -FQAAKDFAT ISTGMFVLGT GY-------S ALK------- ----------  63
TvTim23       ---------- -MQTIRELVD YAPIAEDI-- --GKSFIIGY GAGTACGLVV GFSRNH---- ----------  38
TvTim22-like  ETPIYAELGL GPNPSKMDMA TKVLKKSYHT FQNTALEFSL VYSG-CLIIN DLV------- ----------  58
TvTim23-like  QLEVLKIQLL DEKNYEIRPK -IDIVKEFPF KLTKSV--GI TLGT------ ---------- ----------  49
TvTim17-like  ---------- ---------M QRPTFIEYAQ RTGRGMITGH GFALMGSMKH ALF------- ----------  34
Tim17         ---------- MSADHSRDPC PIVILNDF-- --GGAFAMGA IGGVVWHGIK GFR------- ----------  39
Tim23         LDLEEEQLSS LEGSQGLIPS -RGWTDDLCY GTGAVYLLGL GIGGFSGMMQ GLQ------- ---------- 122
Tim22         TPEEQG---E RGAEMIMNFM TSCPGK-SVV SGVTGFALGG VLGLFMASMA YDTPLHTPTP ANTAATATAG  88

                                       H7            TM2

TvTim22       ---------- -NLPVFNHGK PFSVKSTL-- --KEGIQIGV SLGAQAALTN LCETTLAIYR GQQKFYDP-- 116
TvTim23       ---------- DNLPL----- -ITLNDTLNC IEKYSGEFGY TFGIAAGLHT L---SCQLSK NLKPIQKH--  90
TvTim22-like  ---------- DKKP------ -IQWRNNI-- --VTATTYSL NIATSLALAE IINDSIALHR GERKIYDS-- 105
TvTim23-like  ---------- -STLLTAAAS GFAMRNIK-- --SVKEDLGL AIPVFG-MFS GIDFAVNYTL TKSFGYKNPT 103
TvTim17-like  ---------- -EAPYGSKMA EFKSNFAL-- ---TSKQNAV SMAKWALCSA TITPYITS-R IKNPIVAD--  85
Tim17         ---------- -NSPLGERGS -GAMSAIK-- --ARAPVLGG NFGVWGGLFS TFDCAVKAVR KREDPWNA--  91
Tim23         ---------- -NIPPNSPGK -LQLNTVLNH ITKRGPFLGN NAGILALSYN IINSTIDALR GKHDTAGS-- 178
Tim22         NIGVGGISRT VQQISDLPFR QQMK-LQFTD MGKKSYSSAK NFGYIGMIYA GVECVIESLR AKNDIYNG-- 155

                   TM3                         TM4

TvTim22       ----IIAGAA VGATLNCYKG ---FKQMGIG AAKGATLAVF LV----TAEA VSDRF----- ---------- 160
TvTim23       ----AIAGGV AGTFIGSHWG ---MKGAIGG GVVGAALGA- ------GYGY ASTNPELLKY FTK------- 137
TvTim22-like  ----IIASTI SGGIVEIPHG ---ASAVYRG AVSGALWSSA MA----GMQY VFSKFLSDKP DSENK----- 155
TvTim23-like  QPICIVSGAT AGAACGYYFG NKKLKPTLFG GIAGGIYGAI RN-------- TPMNLLGFEP F--------- 156
TvTim17-like  ----IAEGSM VGALLEWRNG ---MKGIASG AFQGALQSVF MTVVGKGLQI VLTPINTYRN NQRVKKFTIE 148
Tim17         ----IIAGFF TGGALAVRGG ---WRHTRNS SITCACLLGV IE----GVGL MFQRYAAWQA KPMAPPLPEA 150
Tim23         ----IGAGAL TGALFKSSKG ---LKPMGYS SAMVAAACAV WC-------S VKKRLLE--- ---------- 221
Tim22         ----VTAGFF TGAGLAYKAG ---PQAALMG GAGFAAFSAA ID-------- LYMKSEDGRP P--------- 201

TvTim22       ---------- ---------- -- 160
TvTim23       ---------- ---------- -- 139
TvTim22-like  ---------- ---------- -- 159
TvTim23-like  ---------- ---------- -- 156
TvTim17-like  RNQNTLKSPF EAISTVFFNS PK 170
Tim17         PSSQPL---- ---------- QA 158
Tim23         ---------- ---------- -K 222
Tim22         --QNDF---- ---------- KE 207
```

Figure S2. Phylogenetic analysis of Tim17 family proteins. The maximum likelihood (ML) tree was constructed using IQ-TREE (Best fit; LG+C50+G4 model with 91 sequences and 100 sites). Ultra-fast bootstrap values were calculated using 10000 replicates.

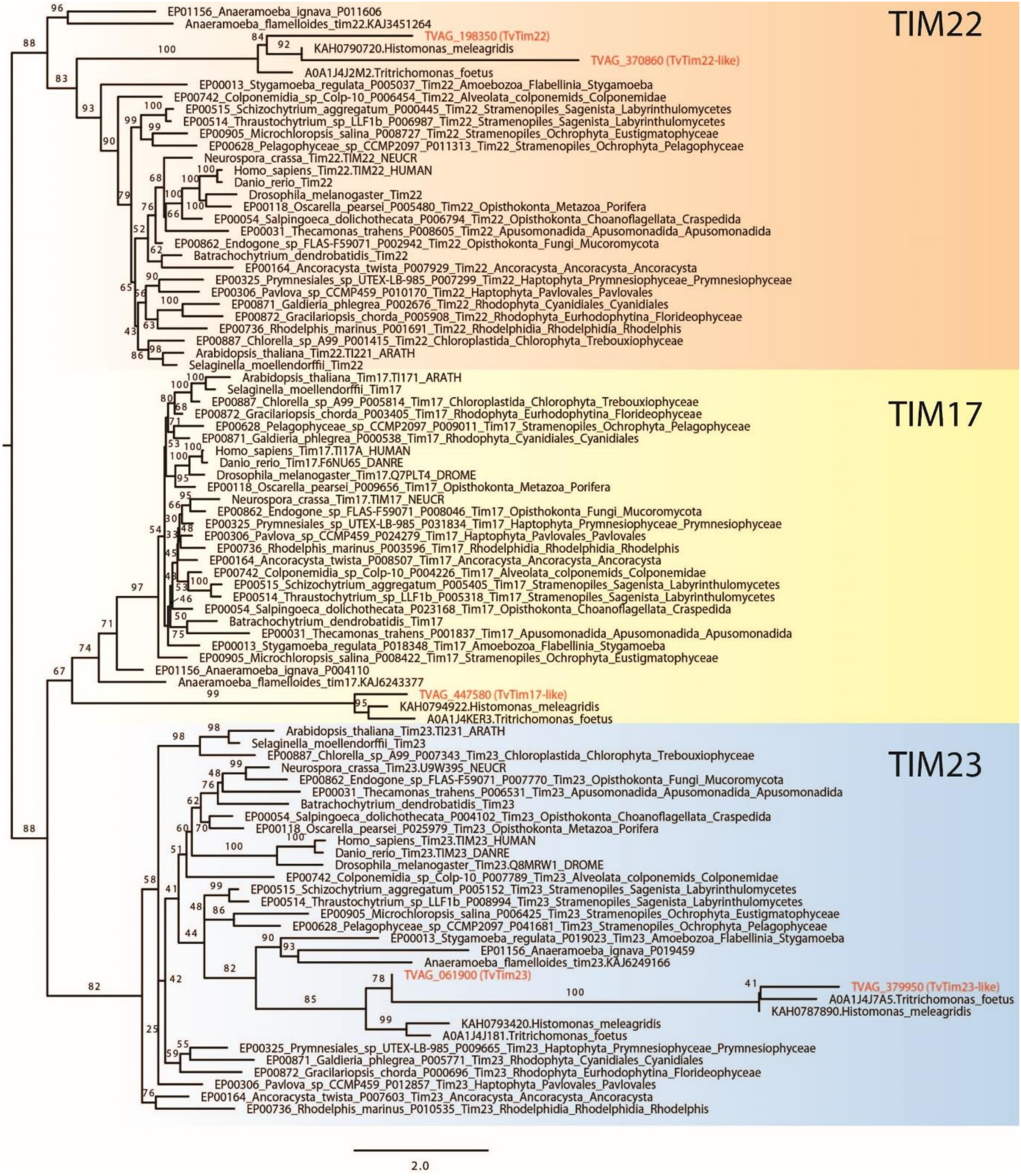

Figure S3. Phylogenetic analysis of Tim17 family proteins in parabasalids

The maximum likelihood (ML) tree was constructed using IQ-TREE (Best fit; LG+ G4 model with 34 sequences and 149 sites). Non-parametric bootstrap values were calculated using 1000 replicates.

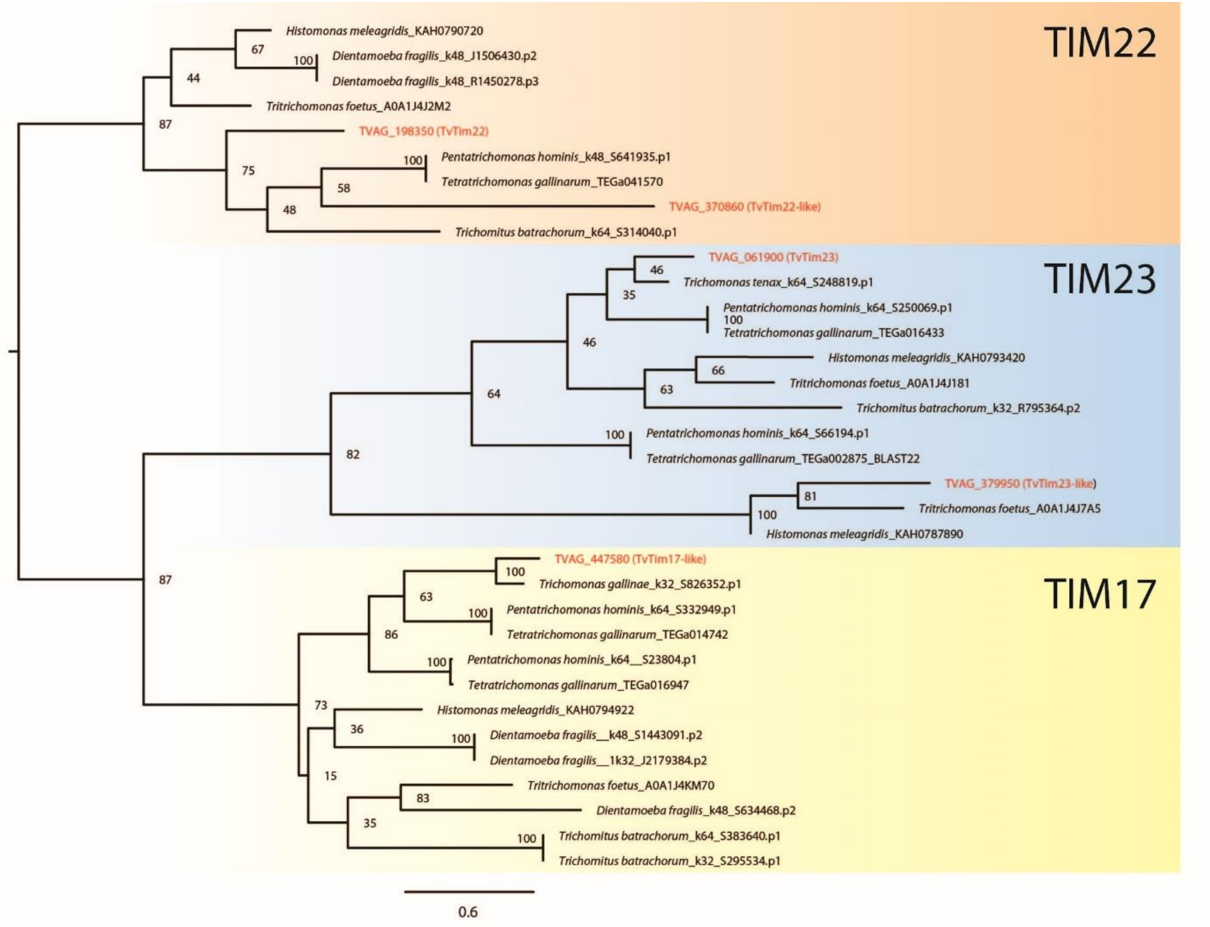

Figure S4. Analysis of immunoprecipitated TIM/TvTim22 complex. The protein distribution was analyzed by quantitative MS analysis (Additional file 3: Table S2). Proteins separated by SDS-PAGE were stained with Silver Stain Kit (Pierc, left) and Coomassie Brilliant Blue G-250 (CBB, right).

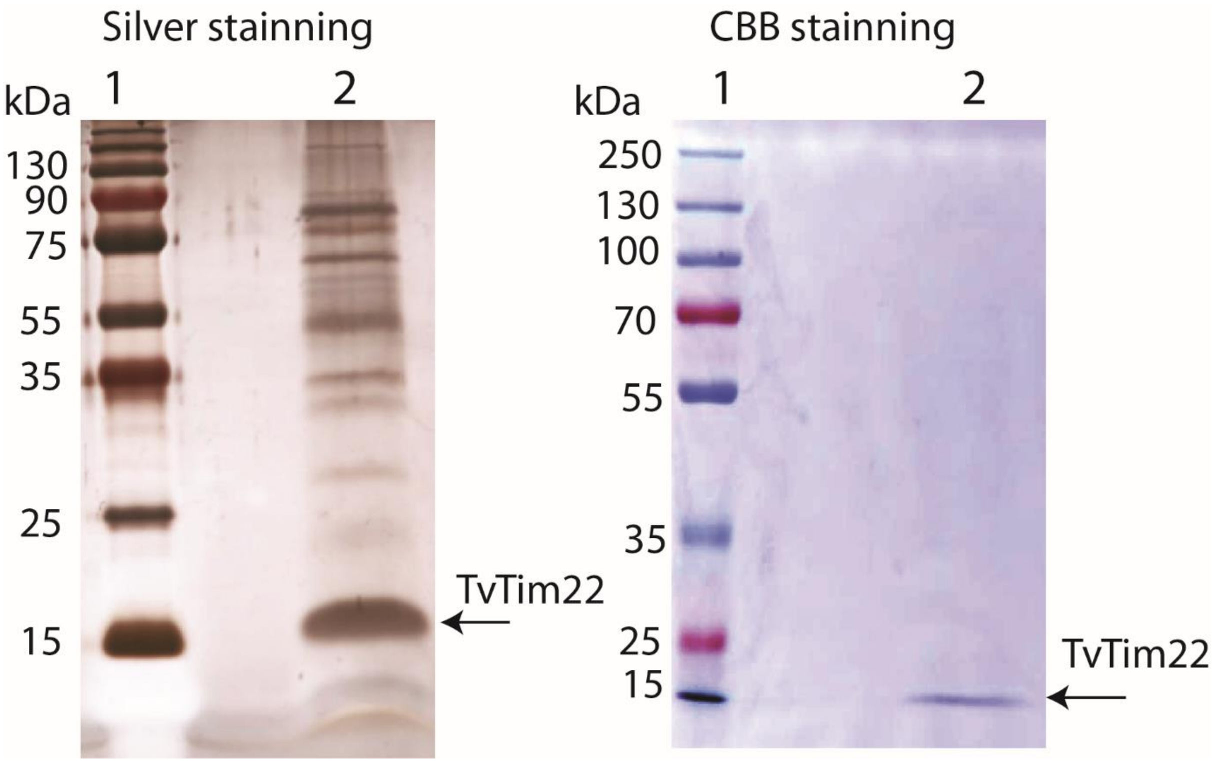

Figure S5. Unprocessed cryo-electron microscopy of single particle TvTIM/TvTim22 complex. TvTIM particles tended to stick outside of the observable areas. Grids preparation techniques to overcome this tendency were done without success e.g adjusting glow charged time, changing grids type/grids with carbon supportive layer, etc. Hence, the datasets were not suitable for high throughput imaging and single particle analysis. Red circles indicate TvTIM/TvTim22 particles. Cryo-electron microscopy was performed as described [8]. Briefly, the 3 μl sample drop was applied to freshly glow-discharged transmission electron microscopy grids (Protochips, Cu, 300 mesh, R1.2/1.3) and vitrified into liquid ethane using ThermoScientific Vitrobot Mark IV (4 °C, 100% rel. humidity, 30 s waiting time, 3 s blotting time). The grids were subsequently mounted into the Autogrid cartridges and loaded into a Talos F200C microscope (ThermoScientific).

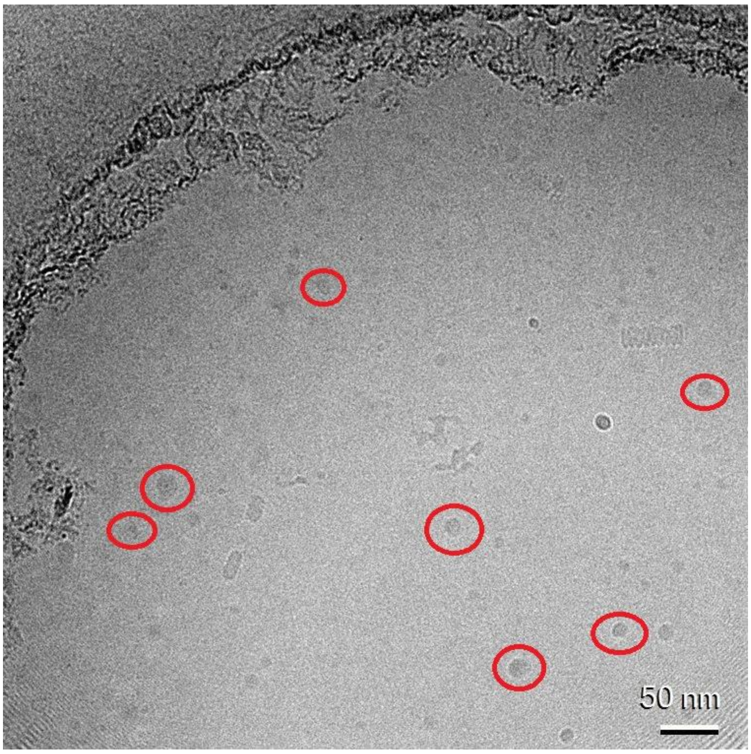

Figure S6. Heat map of protein quantity (LFQ values) coIPed with TvTim22, TvTim22-like, TvTim23, TvTim23-like, TvTim17-like as baits using crosslinker, or without crosslinker (TvTim22 Native). Each experiment was performed in at least triplicates. wt, wild-type cells.

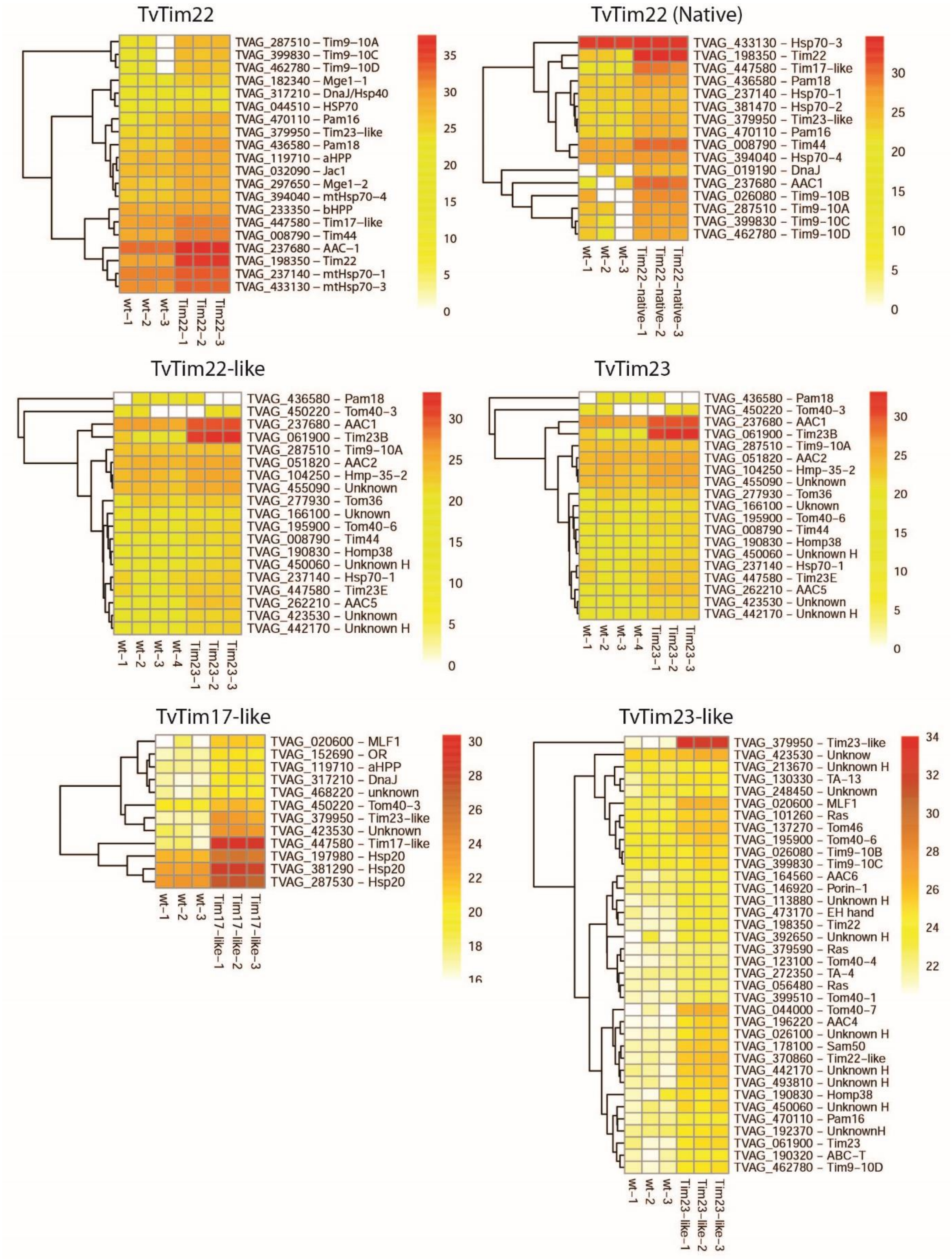

Figure S7. Cell localization of TvTim22 and Tim44. (A) Standard confocal immunofluorescence microscopy. *T. vaginalis* expressed a recombinant HA-tagged TvTim22 (in green, AlexaFluor 488) and V5-tagged Tim44 (in red, AlexaFuor 594) under the control of α-succinyl CoA synthetase (SCS) promotor. The nucleus is labeled with DAPI (4′, 6-diamidino-2-phenylindole). DIC, differential interference contrast. (B) Expansion immunofluorescence microscopy. *T. vaginalis* expressed a recombinant HA-tagged TvTim22 (in green, AlexaFluor 488) and V5-tagged Tim44 (in violet, AlexaFluor 647) under the control of SCS promotor. The cell was visualized using N-hydroxysuccinimidyl(NHS)-ester (brown) at 550 nm. White arrows indicate Tim44 and TvTim22 patches.

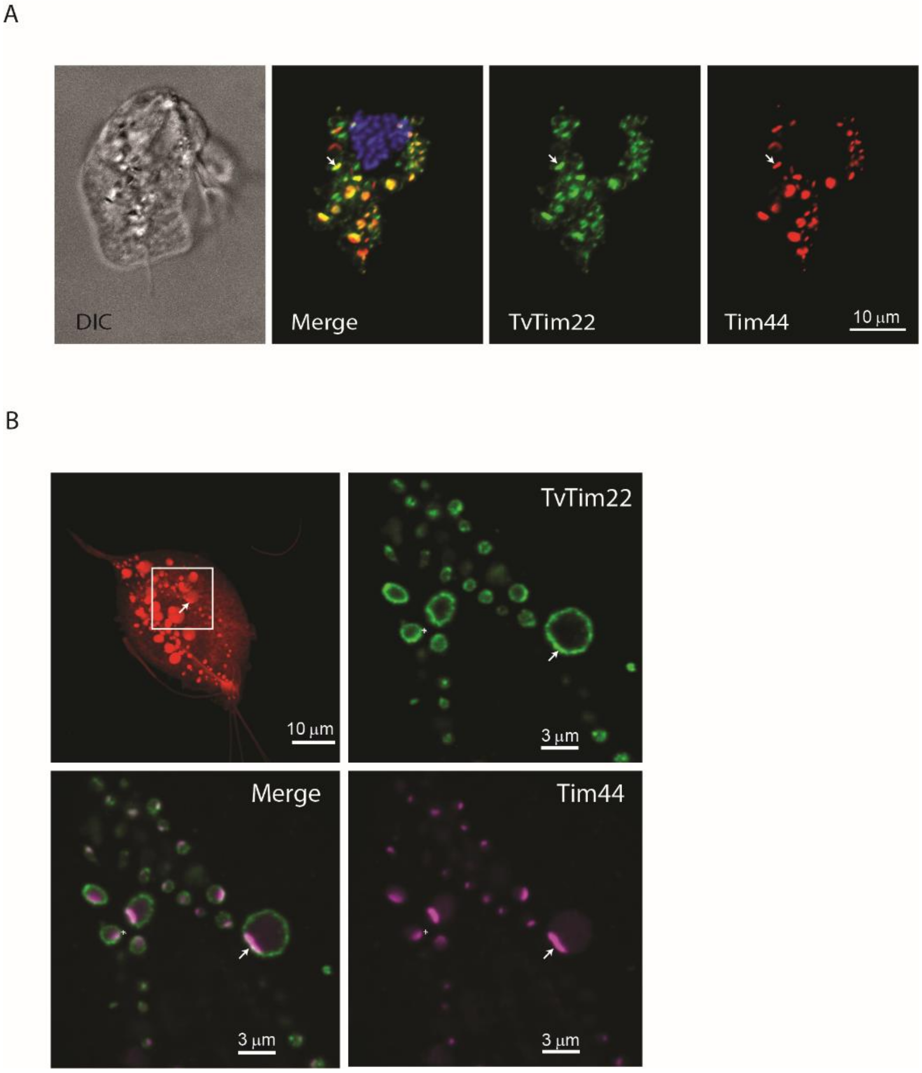

Figure S8. Full immunoblots presented in Figures 4C and 4E.

Western blot analysis of cell lysate (Lys), isolated hydrogenosomes (Hyd), immunoprecipitated fraction (IP), and unbound fraction (Ub). Red rectangles indicate cropped areas.

Tim44-HA bait; anti-HA antibody

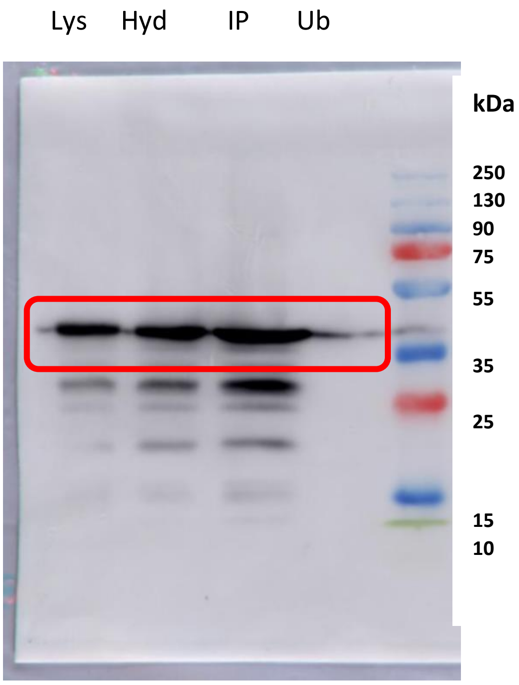

AAC1-HA bait; anti-HA antibody

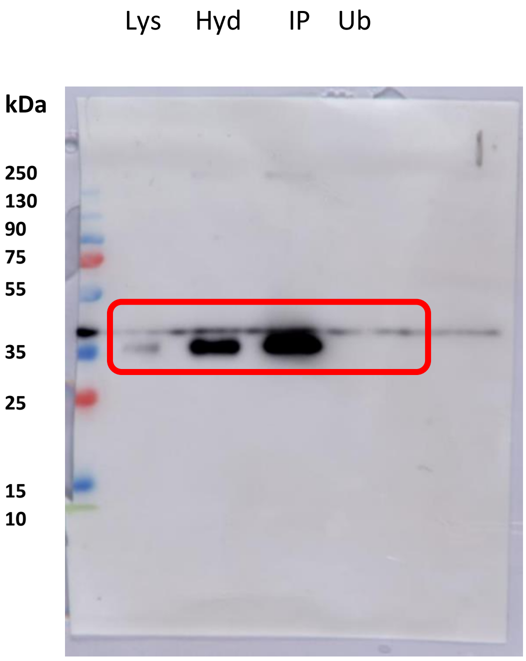

Left: Tim22-V5, anti V5 Ab (Tim44-HA bait) Right: Tim22-V5, anti V5 Ab (AAC1-HA bait)

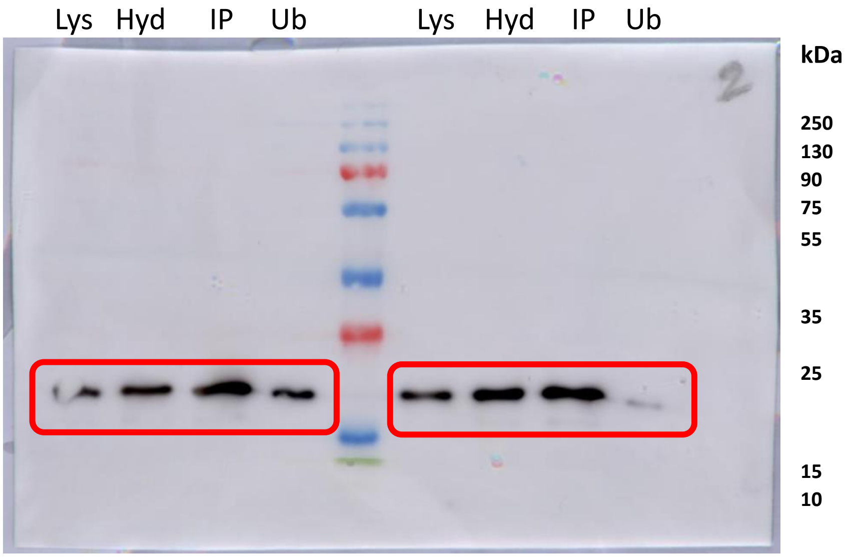

Left: Anti-Sam50 Ab (Tim44-HA bait) Right: Anti-Sam50 Ab (AAC1-HA bait)

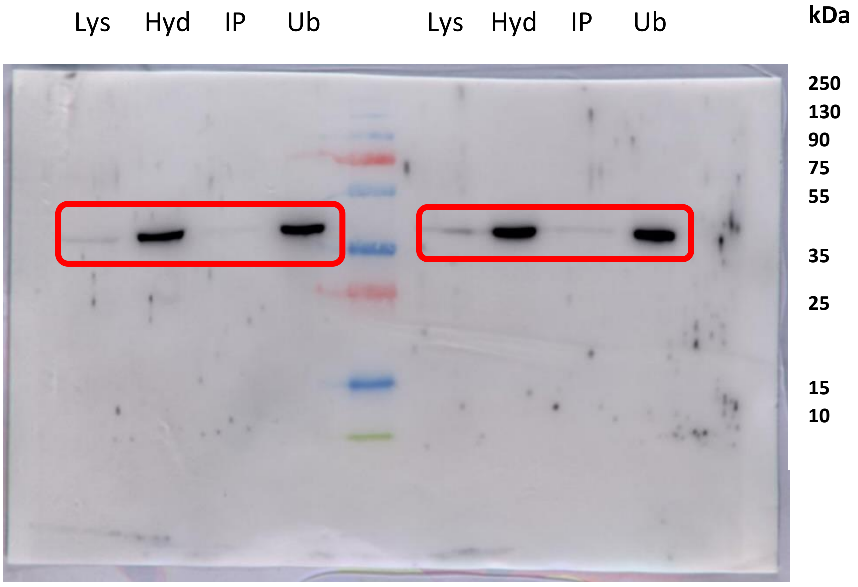

Figure S9. Full autoradiograms presented in Figure 5. Red rectangles indicate cropped areas.

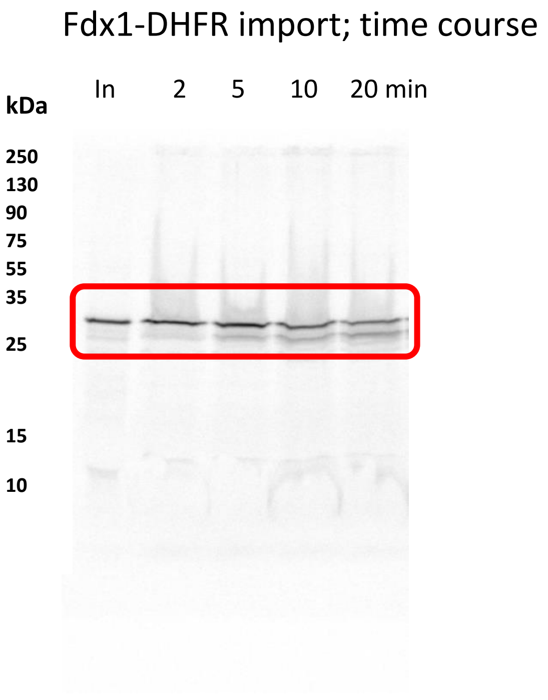

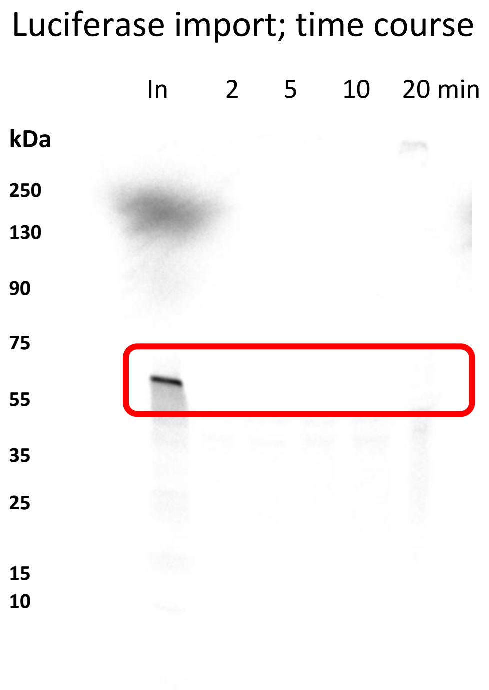

Figure S10. *In vitro* import of AAC1 into *T. vaginalis* hydrogenosomes. Time-course of radiolabeled AAC1- DHFR import into isolated hydrogenosomes *in vitro*. (B) Autoradiography of AAC1-DHFR that was coIP-ed using HA-tagged TvTim22 as bait. MTX, methotrexate

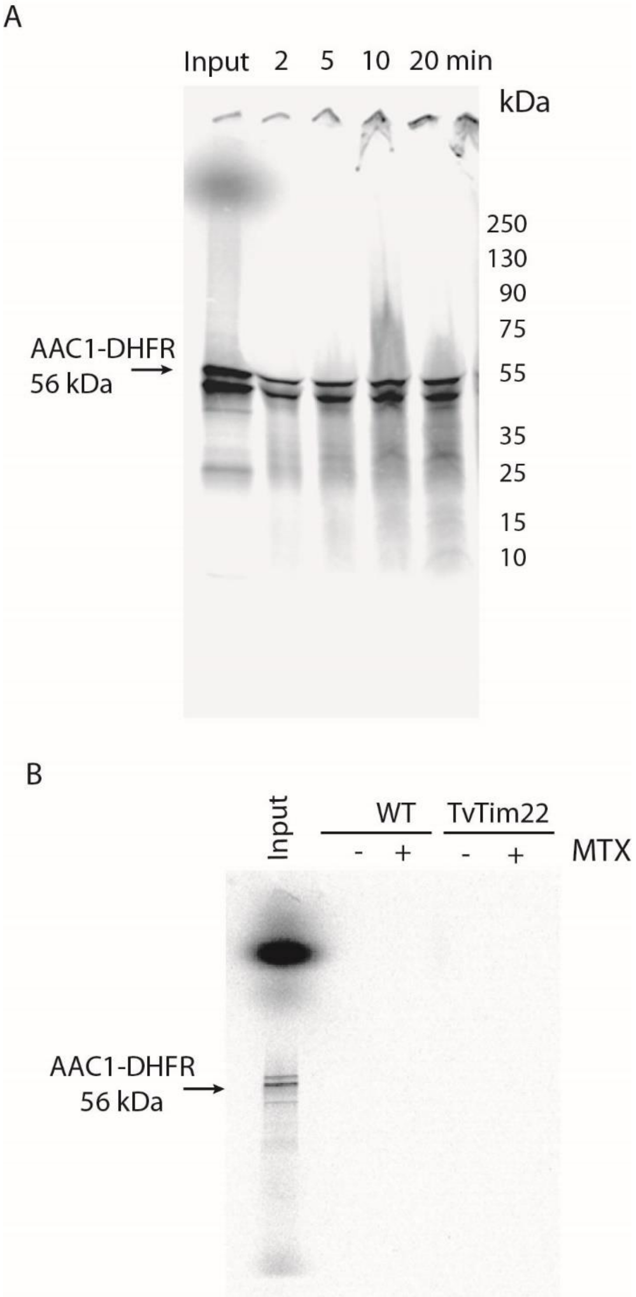

References

1. Demishtein-Zohary K, Günsel U, Marom M, Banerjee R, Neupert W, Azem A, et al. Role of Tim17 in coupling the import motor to the translocation channel of the mitochondrial presequence translocase. Elife. 2017;6:e22696.

2. Demishtein-Zohary K, Marom M, Neupert W, Mokranjac D, Azem A. GxxxG motifs hold the TIM23 complex together. FEBS J. 2015;282:2178–86.

3. Kumar A, Matta SK, D'Silva P. Conserved regions of budding yeast Tim22 have a role in structural organization of the carrier translocase. J Cell Sci. 2020;133: jcs244632.

4. Qi L, Wang Q, Guan Z, Wu Y, Shen C, Hong S, et al. Cryo-EM structure of the human mitochondrial translocase TIM22 complex. Cell Res. 2021;31:369–72.

5. Zhang Y, Ou X, Wang X, Sun D, Zhou X, Wu X, et al. Structure of the mitochondrial TIM22 complex from yeast. Cell Res. 2021;31:366–8.

6. Zhou S, Ruan M, Li Y, Yang J, Bai S, Richter C, et al. Solution structure of the voltage-gated Tim23 channel in complex with a mitochondrial presequence peptide. Cell Res. 2021;31:821–4.

7. Meier S, Neupert W, Herrmann JM. Conserved N-terminal negative charges in the Tim17 subunit of the TIM23 translocase play a critical role in the import of preproteins into mitochondria. J Biol Chem. 2005;280:7777–85.

8. Dubochet J, Adrian M, Chang JJ, Homo JC, Lepault J, McDowall AW, Schultzet P. Cryo-electron microscopy of vitrified specimens Q. Rev. Biophys., 1988; 21:129-228.
