# Supplementary material for: A hybrid TIM complex mediates protein import into hydrogenosomes of Trichomonas vaginalis
Source: BMC Biol. 2024 Jun 3;22:130. doi: 10.1186/s12915-024-01928-8 (PMC11145794; doi:10.1186/s12915-024-01928-8)
Supplement: Supplementary file 2 — Additional file 2:Table S1. Summary of phylogenetic inferences, homology, and structural predictions. [file 12915_2024_1928_MOESM2_ESM.docx]

**Additional file 2: Table S1.** Summary of phylogenetic inferences, homology searches, and structure predictions

| Accession  Number | Phylogeny*  inference | PSI BLAST  NCBI** | Hhpred against COG KOG  (probabilities) | HMMER***  Top Hit | Hhsearch pairwise (probabilities) | Name |
| --- | --- | --- | --- | --- | --- | --- |
| TVAG_198350 | Tim22 | *T. vaginalis* TVAG_370860, 2e-5  *T. foetus* OHS93688.1, 3e-67  *H. meleagridis* KAH0790720.1, 1e-60  ***A. flamelloides* Tim22, KAJ3430941; 0,009** | KOG1652 Tim17: 98,42  **KOG3225 Tim22: 98,8**  KOG3324 Tim23: 98,7  KOG4608 Timmdc1^+^: 98,67 | OHS93688.1  A0A1J4J2M2  *T. foetus*  2.6e-88 | TVAG_198350: 100  TVAG_061900: 80,4  **TVAG_370860: 98,01**  TVAG_379950: 5,38  TVAG_447580: 96,3 | TvTim22 |
| TVAG_061900 | Tim23 | *T. foetus*, OHS93304.1, 5e-50  *H. meleagridis,* KAH0793420.1, 4e-36  ***A. flamelloides* KAJ6229560, Tim23, 4e-07** | KOG1652 Tim17: 98,5  KOG3225 Tim22: 98,39  **KOG3324 Tim23: 98,77**  KOG4608 Timmdc1^+^: 98,45 | OHS93304.1  A0A1J4J181  *T. foetus*  1.4e-83 | **TVAG_198350: 80,53**  TVAG_061900: 100  TVAG_370860: 2,61  TVAG_379950: 75,62  TVAG_447580: 69 | TvTim23 |
| TVAG_370860 | Tim22 | *T. vaginalis,* TVAG_198350, 3e-09  *T. foetus* , OHS93688.1, 8e-19  *H. meleagridis,* KAH0790720.1, 2e-38 | KOG1652 Tim17: 91,07  KOG3225 Tim22: 75,29  KOG3324 Tim23: 83,93  **KOG4608 Timmdc1^+^: 93,9** | OHS93688.1  A0A1J4J2M2  *T. foetus*  0.00047 | **TVAG_198350: 98,01**  TVAG_061900: 2,61  TVAG_370860: 100  TVAG_379950: 0,1  TVAG_447580: 27,95 | TvTim22-like |
| TVAG_379950 | Tim23 | *T. foetus* OHS95110.1, 7e-80  *H. meleagridis,* KAH0787890.1, 2e-81 | KOG1652 Tim17: 50,33  KOG3225 Tim22: 48,54  KOG3324 Tim23: 53,55  KOG4608 Timmdc1^+^: 63,55 | OHS95110.1  A0A1J4J7A5  *T. foetus*  2.4e-90 | TVAG_198350: 5,38  **TVAG_061900: 75,62**  TVAG_370860: 0,1  TVAG_379950: 100  TVAG_447580: 4,68 | TvTim23-like |
| TVAG_447580 | Tim17 | *T. foetus* OHT09424.1, 8e-74  *H. meleagridis* KAH0794922.1, 2e-74  ***A. flamelloides* Tim17, KAJ3449200, 0.005** | **KOG1652 Tim17: 97,85**  KOG3225 Tim22: 97,01  KOG3324 Tim23: 97,06  KOG4608 Timmdc1^+^: 97,49 | OHT12032.1  A0A1J4KM70  *T. foetus*  5.1e-82 | **TVAG_198350: 96,3**  TVAG_061900: 69  TVAG_370860: 27,95  TVAG_379950: 4,68  TVAG_447580: 100 | TvTim17-like |

* According to Figure 1B and Additional file1: Figure S2

**PSI-BLAST (2 iterations; 1e-3 eval cutoff) against the NCBI nr database (e-values are shown below each hit (637 497 959 sequences, update date 2023/12/04)

^+^ A metazoan-specific divergent paralog of Tim23 [1]

***HMMER [2]: Alphafold_uniprot50_Aug22 (<https://toolkit.tuebingen.mpg.de/tools/hmmer>)

1. Žárský V, Doležal P. Evolution of the Tim17 protein family. Biol Direct. 2016;11:54.

2. Zimmermann L, Stephens A, Nam SZ, Rau D, Kübler J, Lozajic M, Gabler F, Söding J, Lupas AN, Alva V. J Mol Biol. 2018 Jul 20. S0022-2836(17)30587-9.
