# Supplementary material for: A hybrid TIM complex mediates protein import into hydrogenosomes of Trichomonas vaginalis
Source: BMC Biol. 2024 Jun 3;22:130. doi: 10.1186/s12915-024-01928-8 (PMC11145794; doi:10.1186/s12915-024-01928-8)
Supplement: Supplementary file 4 — Additional file 4:Table S3. Parameters of representative classes of TIM/TvTim22 particles selected from a dataset of electron microscopy images. [file 12915_2024_1928_MOESM4_ESM.doc]

Additional file 4: Table S3. Parameters of the representative classes of TIM particles selected from a dataset of electron microscopy images.

| Class No. | Diameter of the pore structure | Area of the pore structure | Pore size | Adjacent mass |  | Total area |  |  |  |
| --- | --- | --- | --- | --- | --- | --- | --- | --- | --- |
|  | [nm] | [nm^2^] | [nm] | [nm^2^] |  | [nm^2^] |  |  |  |
| 1 | 7.275 | 36.110 | 3.328 | 28.372 |  | 64.482 |  |  |  |
| 2 | 8.305 | 37.857 | 2.995 | 106.083 |  | 143.940 |  |  |  |
| 3 | 7.398 | 46.667 | 5.408 | 31.617 |  | 78.294 |  |  |  |
| 4 | 6.875 | 36.859 | 3.494 | 39.438 |  | 76.297 |  |  |  |
| 5 | 6.541 | 36.692 | 3.744 | 50.421 |  | 87.113 |  |  |  |
| 6 | 6.977 | 32,948 | 3.245 | 78.793 |  | 114.986 |  |  |  |
| 7 | 7.388 | 33.863 | 3.162 | 31.450 |  | 65.313 |  |  |  |
| 8 | 7.388 | 36.859 | 3.078 | 52.168 |  | 89.027 |  |  |  |
| Average | 7.26 | 34.08 | 3.36 | 52.29 |  | 89.93 |  |  |  |
